# Viral‐Directed Augmentation of Kupffer Cell Cross‐Presentation Provokes Antitumor Immunity Against Liver Metastasis

**DOI:** 10.1002/advs.202504929

**Published:** 2025-07-29

**Authors:** Chen Chen, Qing Zhang, Jiajia Li, Xia Zhou, Daxing Gao, Lu Li, Dabing Huang, Jizhou Wang, Zhutian Zeng

**Affiliations:** ^1^ State Key Laboratory of immune response and immunotherapy Department of Oncology The First Affiliated Hospital of USTC Center for Advanced Interdisciplinary Science and Biomedicine of IHM Division of Life Sciences and Medicine University of Science and Technology of China Hefei Anhui 230001 China; ^2^ Department of Hepatobiliary Surgery Center for Leading Medicine and Advanced Technologies of IHM Division of Life Sciences and Medicine University of Science and Technology of China Hefei Anhui 230001 China

**Keywords:** cross‐presentation, intravital microscopy, Kupffer cell, liver metastasis, oncolytic virus, type I IFN, VSV

## Abstract

Liver metastasis is associated with poor prognosis and resistance to immune checkpoint inhibitors. Functional modulation of Kupffer cells (KCs) holds promise as an alternative immunotherapeutic approach. Leveraging their capacity to capture circulating virions, an oncolytic virus‐based KC‐targeting strategy is developed that demonstrated efficacy and safety in treating multifocal liver metastasis. A single intravenous infusion of the M51R mutant vesicular stomatitis virus (VSV‐M51R), but not wild‐type (WT) VSV, induced significant tumor regression in mouse models of forced liver metastasis, independent of direct oncolysis. The ineffectiveness of VSV‐WT is attributed to its induction of massive KC apoptosis, whereas VSV‐M51R replicated transiently within KCs without compromising viability. Instead, VSV‐M51R promoted KC proliferation in tumor‐adjacent areas, enhancing their access to tumor foci and cross‐presentation of tumor antigens. This led to robust activation of hepatic anti‐tumor CD8^+^ T‐cell responses, which required mitochondrial antiviral signaling protein‐dependent type I interferon triggering in KCs. Depletion of KCs abolished the T cell stimulating and anti‐tumor effects of VSV‐M51R. Furthermore, simultaneous blockade of programmed cell death‐ligand 1(PD‐L1) during VSV‐M51R treatment achieved remarkable synergistic efficacy in treating monotherapy‐resistant late‐stage liver metastasis. These findings underscore the pivotal role of KCs in systemic oncolytic virotherapy and offer a potentially applicable strategy for treating advanced liver metastasis.

## Graphical abstract

Targeting Kupffer cells (KC) with a single intravenous infusion of the oncolytic virus VSV‐M51R rather than VSV‐WT induces effective tumor regression in various types of liver metastatic cancers in mice. VSV‐M51R promotes KC proliferation and enhances their antigen cross‐presentation capacity without compromising viability, leading to the induction of robust tumor antigen‐specific CD8^+^ T cell responses that can be further potentiated by anti‐PDL1 treatment.

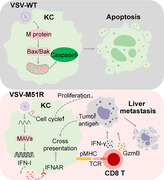

## 1 Introduction

Approximately 90% of cancer‐related deaths are attributed to metastasis, a process in which cancer cells spread from primary tumors and prosper in distal organs. The liver serves as the primary site for the seeding and growth of hematogenously disseminated cancer cells, particularly those originating from the gastrointestinal tract. Liver metastasis is generally associated with poor prognosis and typically manifests as multifocal, making surgical resection difficult.^[^ ^1^, ^2^ ^]^ Owing to the inherent tolerogenic properties of the liver, liver metastases are also resistant to T‐cell‐based immune checkpoint inhibitors,^[^ ^3^, ^4^, ^5^ ^]^ underscoring the urgent need to develop alternative immunotherapeutic approaches.

Kupffer cells (KCs) are the most abundant immune cells in the liver. These embryonically derived macrophages reside intravascularly to ensure prompt immunosurveillance against blood‐borne particles, including circulating tumor cells.^[^ ^6^, ^7^, ^8^ ^]^ In addition to directly phagocytosing metastatic tumor cells via C‐type lectin receptors and Fc receptors,^[^ ^9^, ^10^, ^11^ ^]^ KCs also orchestrate early antitumor innate immune responses, collectively restricting the growth of liver‐seeded metastatic tumors.^[^ ^12^, ^13^ ^]^ However, these antitumor functions of KCs are compromised as metastases progress, likely due to their reduced accessibility to tumor foci or repolarization toward an anti‐inflammatory state.^[^ ^14^, ^15^, ^16^ ^]^ The functional reprogramming of KCs has emerged as an encouraging immunotherapeutic strategy for treating liver malignancies. Our previous study demonstrated that targeted disruption of MafB and cMaf expression in KCs could expand peritumoral KCs and unleash their tumoricidal capacity, leading to durable T‐cell immunity against liver metastasis.^[^ ^15^ ^]^ Similarly, recent reports indicate that immune stimuli, such as IFN‐α or β‐glucan, can target KCs to induce T‐cell‐dependent tumor regression.^[^ ^17^, ^18^ ^]^ These findings suggest a role of KCs in reinvigorating antitumor T‐cell immunity, which underlies the efficient control of liver metastases. Although KCs are typically considered tolerogenic antigen‐presenting cells (APCs) that favor the induction of T‐cell tolerance,^[^ ^19^ ^]^ efficient T‐cell priming by KC‐mediated cross‐presentation has been observed during acute viral infections,^[^ ^20^ ^]^ suggesting the possibility of harnessing viral stimulation to subvert the tolerogenic properties of KCs and provokes effective T‐cell immunity against liver metastasis.

Oncolytic viruses (OVs) offer a promising alternative strategy in cancer therapies because of their inherent ability to selectively kill tumor cells. Local delivery of OVs results in immunogenic cell death in tumor cells, yielding significant therapeutic benefits in cancer patients.^[^ ^21^, ^22^ ^]^ However, intratumoral administration of OVs is technically challenging and less effective for treating disseminated metastatic cancers, such as liver metastases, which are often multifocal and contain some tumor lesions that are too small for injection. While systemic administration of OVs appears to be a more rational approach for metastatic cancers, exposure to the bloodstream inevitably increases the risk of viral sequestration and inactivation by the immune system, leading to reduced viral entry into tumor foci and impaired oncolysis.^[^ ^23^ ^]^ It was recently reported that infection of noncancerous cells is also effective in promoting T‐cell‐dependent tumor regression egression following intravenous OV delivery, although the exact cell type that triggers antitumor immunity in response to OVs remains elusive.^[^ ^24^ ^]^ In fact, KCs are the predominant phagocytes capable of taking up the majority of circulating viral particles.^[^ ^25^, ^26^, ^27^ ^]^ Although this filtering function potentially impedes the direct oncolytic effects of OVs, the selective entrapment of OVs in KCs could be leveraged to spur the antitumor properties of these immune sentinels, leading to effective control of metastatic tumors independent of oncolysis. However, given that KCs are prone to undergo rapid cell death during various types of viral infections,^[^ ^26^, ^28^, ^29^ ^]^ a suitable viral platform that skews KCs into immunostimulatory macrophages while maintaining their integrity is necessary.

In this study, we tested the hypothesis of diverting OVs to target and functionally reprogram KCs for the treatment of multifocal liver metastasis using vesicular stomatitis virus (VSV). VSV is a prototypical negative‐strand RNA virus and represents one of the most frequently used OVs in both preclinical models and clinical trials. Owing to the low efficiency of VSV in infecting human cells and its high sensitivity to type I interferons (IFN‐α/β), it is relatively tolerable when applied systemically.^[^ ^30^, ^31^ ^]^ More importantly, VSV can induce strong CD8^+^ T‐cell responses by activating APCs; thus, it is widely engineered as a viral vector for vaccination.^[^ ^32^ ^]^ This renders VSV an attractive candidate in our study, aiming at eliciting T‐cell immunity against liver metastasis by targeting KCs, which are the largest APC population in the liver.

## 2 Results

### 2.1 VSV‐M51R Efficiently Controls Liver Metastasis Independent of Direct Oncolytic Activity

**Figure 1:**
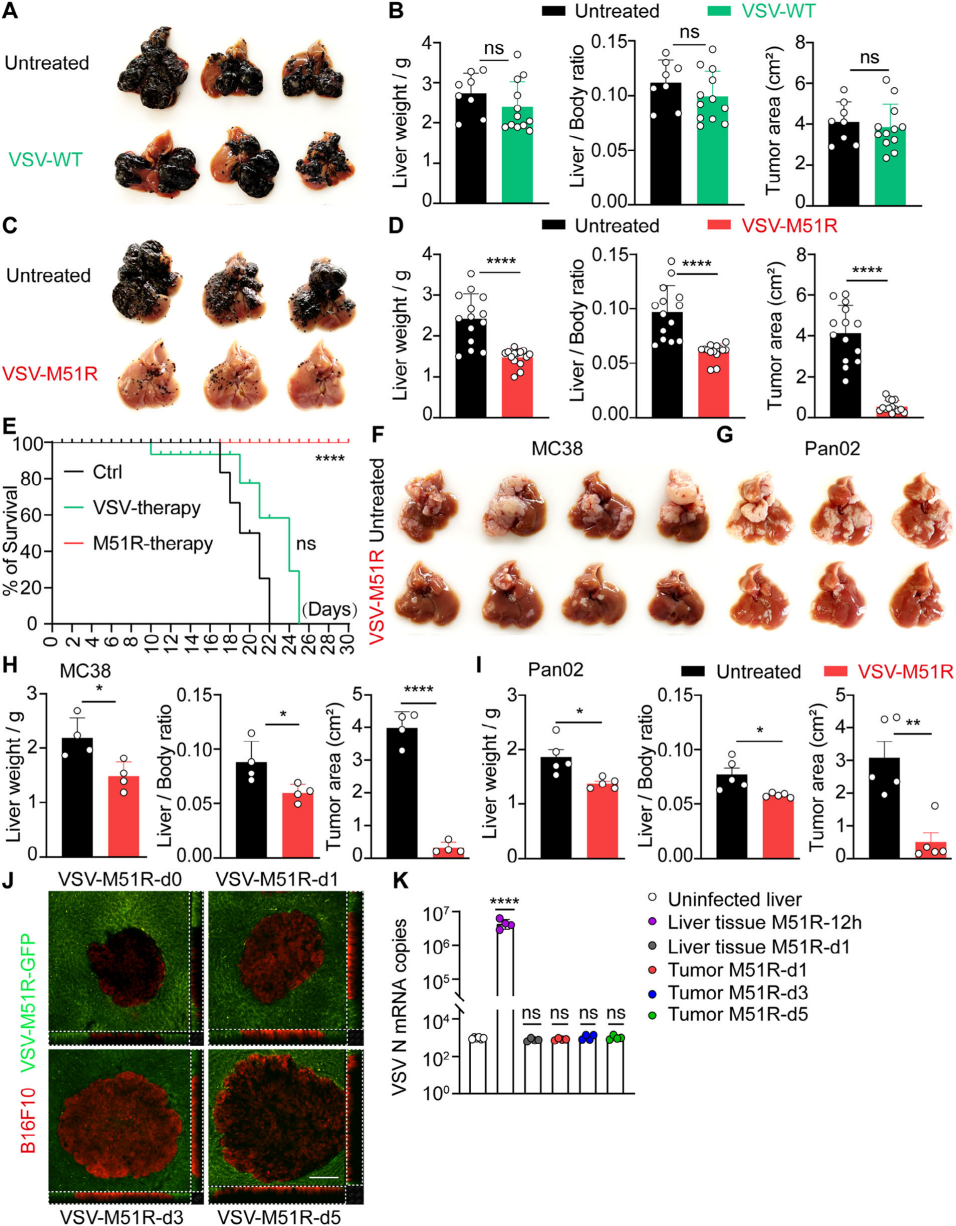
VSV‐M51R efficiently controls liver metastasis independent of its oncolytic activity. A) Mice were treated with 3×10^8^ PFU of VSV on day 7 following B16F10 tumor cell inoculation and were harvested on day 14. B) Quantification of liver weight, the liver‐to‐body weight ratio and the total area of metastatic foci on the liver surface (tumor area) in A. Data from 8–12 mice per group. C) Mice were treated with 3×10^8^ PFU of VSV‐M51R on day 7 following B16F10 tumor cell inoculation and were harvested on day 14. D) Quantification of liver weight, the liver‐to‐body weight ratio and the tumor area in C. Data from 14 mice per group. E) Mouse survival rates were monitored. Data from 10 mice per group. F) Mice were treated with VSV‐M51R on day 7 after MC38 tumor inoculation and harvested on day 14. G) Mice were treated with VSV‐M51R on day 10 after Pan02 tumor inoculation and harvested on day 21. H) Quantification of liver weight, the liver‐to‐body weight ratio and the tumor area in F. Data from 4 mice per group. I) Quantification of liver weight, the liver‐to‐body weight ratio and the tumor area in G. Data from 5 mice per group. J) Two‐photon intravital images of liver metastases from B16F10‐tdTomato tumor‐bearing mice at 12 h and at days 1, 3, and 5 after VSV‐M51R treatment. The X‐Z (bottom) and Y‐Z (right) views for each panel are shown. Scale bar, 100 µm. K) The VSV‐M51R N mRNA copies were quantified by qPCR analysis at 12 h and at days 1, 3, and 5 after VSV‐M51R injection. Data from 3–4 mice per time point. Data are represented as mean ± SEM. ^*^ *p* < 0.05; ^**^ *p* < 0.01; ^***^ *p* < 0.001; ns, no significance. Unpaired Student's t test for **B**, **D**, **H**, and **I**, one‐way ANOVA with Tukey's test for **K**, and two‐sided log‐rank test for **E**.

To evaluate the therapeutic potential of systemic VSV delivery in treating multifocal liver metastases, we employed a well‐established mouse model of forced liver metastasis through intrasplenic injection of highly metastatic B16F10 melanoma cells, which subsequently seed the liver and grow clonally to form multiple metastatic foci. Intravenous administration of 3 × 10^8^ PFU of wild‐type (WT) VSV at 7 days post‐tumor inoculation, when macrometastases had developed in the liver,^[^ ^15^ ^]^ demonstrated only a marginal therapeutic effect. No significant difference in hepatic metastatic tumor burden was observed between the treated and untreated groups (**Figure** **1**A,B).

We initially hypothesized that the limited therapeutic efficacy of VSV‐WT was due to its insufficient immune stimulatory capacity. The M51R mutant VSV (VSV‐M51R) is a naturally occurring variant in which the 51th amino acid (methionine) of the viral matrix (M) protein is replaced with arginine. This mutation abolishes M protein‐mediated inhibition of host protein synthesis, thereby greatly enhancing the immune‐stimulatory properties of the virus.^[^ ^33^ ^]^ In contrast to VSV‐WT, a single intravenous infusion of 3 × 10^8^ PFU of VSV‐M51R significantly retarded the growth of metastatic melanoma in the liver (Figure 1C,D). Direct comparison between VSV‐WT and VSV‐M51R treatment in the same cohorts of tumor‐bearing mice confirmed the superior therapeutic outcomes of VSV‐M51R, as evidenced by substantial reductions in both the number and size of hepatic metastatic tumors (Figure S1A,B, Supporting Information). Importantly, while untreated mice succumbed to metastatic tumor progression within 22 days, mice treated with VSV‐M51R exhibited prolonged survival, extending beyond 30 days. In contrast, VSV‐WT treatment did not confer a significant survival advantage (Figure 1E). Similarly, systemic injection of VSV‐M51R significantly reduced the hepatic tumor burden of metastatic MC38 colorectal cancers and Pan02 pancreatic cancers (Figure 1F–I).

To interrogate whether the observed therapeutic effects of VSV strains depend on their direct oncolytic activity, we monitored viral replication within metastatic tumors at various time points following treatment with recombinant VSV‐WT or VSV‐M51R, both of which encode green fluorescent protein (GFP). While active viral replication, as indicated by GFP expression, was readily observed in VSV‐infected B16F10 melanoma cells in vitro (Figure S1C, Supporting Information), no discernable GFP signal was detected within metastatic tumors at days 1, 3, and 5 after treatment with either VSV‐WT or VSV‐M51R, as assessed by two‐photon intravital microscopy (Figure 1H; Figure S1D, Supporting Information). Consistently, the mRNA copy number of the VSV nucleocapsid (N) gene in tumor tissues was comparable to that in liver tissues from uninfected mice (Figure 1I; Figure S1E, Supporting Information), further confirming the absence of viral replication in metastatic tumors during the course of viral treatment. Notably, although metastatic Pan02 tumors responded to VSV‐M51R virotherapy, these cells were refractory to VSV‐M51R infection in vitro (Figure S1F, Supporting Information), which is consistent with a previous report showing that Pan02 cells are resistant to VSV‐ΔM51‐mediated cell lysis,^[^ ^34^ ^]^ suggesting that virus‐mediated direct oncolysis contributes minimally to tumor regression. Furthermore, surface exposure of calreticulin (ecto‐CRT), a hallmark of immunogenic cell death typically associated with virus‐induced oncolysis of tumor cells,^[^ ^35^ ^]^ was observed in VSV‐M51R‐infected B16F10 cells in vitro but not in metastatic tumor cells isolated from VSV‐M51R‐treated mice (Figure S1G–I, Supporting Information). Taken together, these data suggest that systemic delivery of VSV‐M51R effectively controls liver metastasis through mechanisms independent of tumor cell infection and direct oncolysis.

### 2.2 KC Resistance to Apoptosis Dictates the Therapeutic Efficacy of VSV Virotherapy

**Figure 2:**
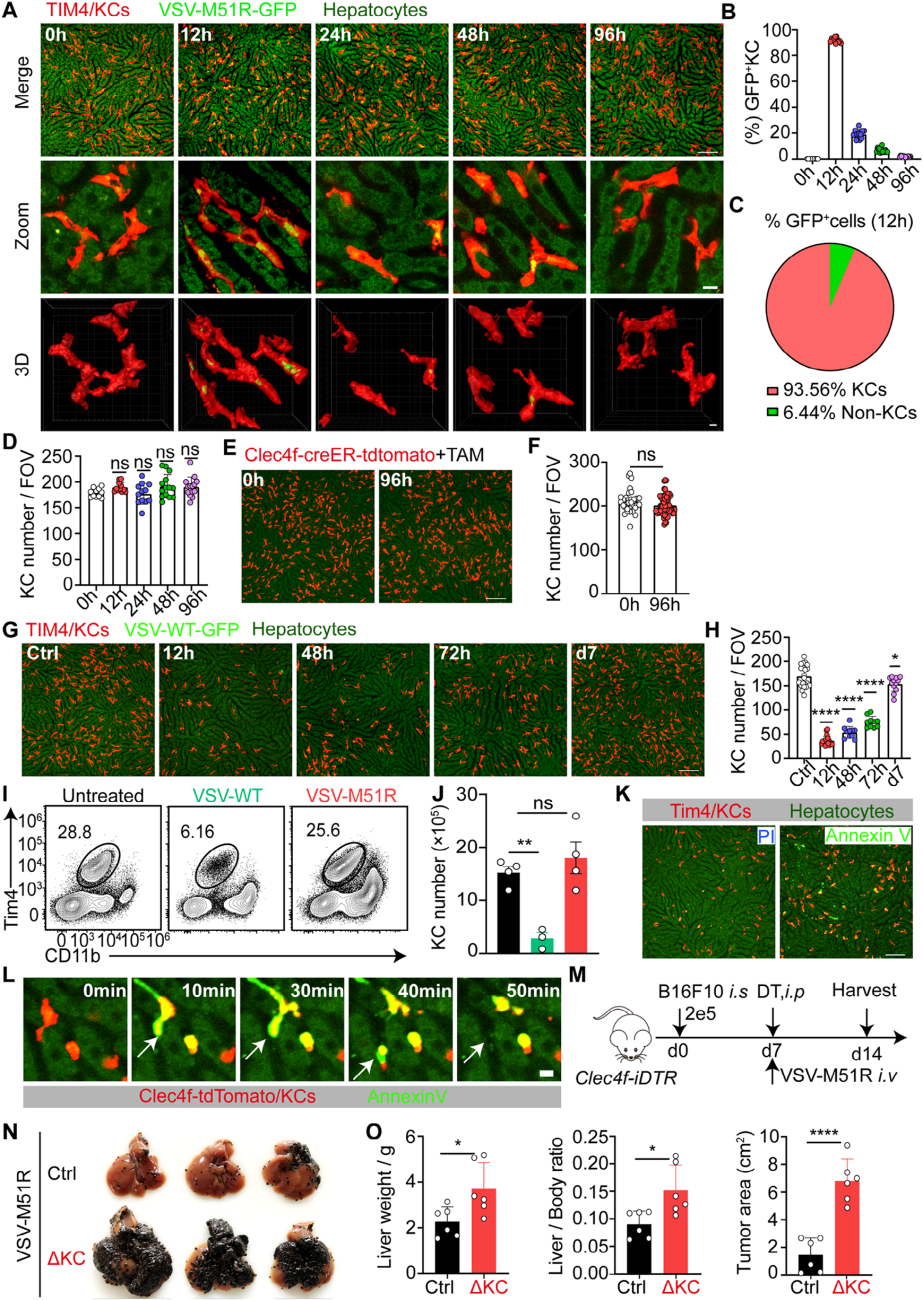
KC resistance to apoptosis dictates the therapeutic efficacy of virotherapy. A) Intravital liver images showing hepatic viral replication at the indicated time points after VSV‐M51R‐GFP treatment. Scale bars: top panel, 100 µm; middle panel: 10 µm; bottom panel: 5 µm. B) Quantification of the ratio of infected KCs per field of view (FOV) in A. C) Quantification of the composition of GFP^+^ cells per FOV at 12 h in A. D) Quantification of KC numbers per FOV in A. E) Intravital images showing resident KCs at 0 and 96 h post‐VSV‐M51R treatment in TAM‐pulsed *Clec4f*‐creER: R26‐LSL‐tdTomato mice. Scale bar, 100 µm. F) Quantification of the number of resident KCs per FOV in E. G) Intravital images showing Tim4‐labeled KCs at the indicated time points after VSV‐WT‐GFP infection. Scale bar, 100 µm. H) Quantification of KC numbers per FOV in G. I) Representative flow cytometric plot of KCs at 12 h after VSV‐WT and VSV‐M51R infection. J) Quantification of TIM4^+^ resident KCs. Data from 3–4 mice per group. K) Representative intravital images of PI and Annexin V staining 6 h post‐VSV‐WT infection. Scale bar, 100 µm. L) Time‐lapse intravital imaging of KCs starting at 6 h post VSV‐WT infection in TAM‐pulsed *Clec4f*‐creER: R26‐LSL‐tdTomato mice. Scale bar, 10 µm. M) Experimental design for N and O. *Clec4f*‐iDTR and control C57BL/6 mice were treated with DT or VSV‐M51R on day 7 following B16F10 tumor inoculation. N) Representative liver photos were taken on day 14. O) Liver weight, the ratio of liver weight to body weight and the tumor area were quantified. Data from 6 mice per group. Data are represented as mean ± SEM. ^*^ *p* < 0.05; ^**^ *p* < 0.01; ^***^ *p* < 0.001; ns, no significance. Unpaired Student's t test for **F** and **O** and one‐way ANOVA with Tukey's test for **B**, **D**, **H**, and **J**. For **B**, **D**, **F**, and **H**, each dot represents one FOV, with FOVs randomly selected from 3–4 mice per group.

The absence of VSV replication in metastatic liver tumors is consistent with previous studies showing that KCs capture and sequester most circulating viral particles,^[^ ^26^ ^]^ leading us to hypothesize that systemically delivered VSV‐M51R may primarily exert its therapeutic effects by targeting KCs. To test this hypothesis, we first tracked hepatic viral distribution by visualizing GFP expression following intravenous injection of VSV‐M51R‐GFP. Intravital microscopy and 3D reconstruction revealed that bright GFP expression was predominantly observed in KCs (**Figure** **2**A). KC expression of virally encoded GFP peaked at 12 h post infection and declined rapidly thereafter, becoming nearly undetectable after 48 h (Figure 2A–C). Similar results were observed in tumor‐bearing mice, where VSV‐M51R replication was largely restricted to KCs (Figure S2A, Supporting Information). Notably, the number of Tim‐4^+^ resident KCs remained unchanged during the course of VSV‐M51R infection (Figure 2A,D). Fate mapping of Clec4f‐creER: R26‐LSL‐tdTomato mice, which enabled permanent labeling of resident KCs upon tamoxifen (TAM) injection, further confirmed that the resident KCs were intact during VSV‐M51R challenge (Figure 2E,F). These data suggest that, upon systemic administration, VSV‐M51R primarily infects KCs in the liver, where the virus undergoes transient replication before being rapidly cleared without compromising the integrity of the resident KC pool.

In sharp contrast to VSV‐M51R treatment, we observed a drastic reduction in Tim‐4^+^ KCs, peaking at 12 h after intravenous injection of VSV‐WT (Figure 2G,H), a time point at which VSV‐M51R displayed the highest replication within KCs. Flow cytometric analysis confirmed a significant decrease in F4/80^+^TIM4^+^ KCs after VSV‐WT treatment (Figure 2I,J), with their numbers not being restored until day 7 post infection (Figure S2B,C, Supporting Information). To understand the mechanism of VSV‐WT‐induced KC loss, we applied Annexin V and propidium iodide (PI) staining in vivo to determine whether KC loss was due to apoptosis or necrosis. The majority of the remaining KCs at 6 h after VSV‐WT injection were stained with Annexin V rather than PI (Figure 2K; Figure S2D, Supporting Information). Time‐lapse intravital imaging revealed that Annexin V^+^ apoptotic KCs frequently underwent fragmentation, and the resulting cell debris was rapidly flushed away from the bloodstream, leading to KC loss (Figure 2L). Consistently, pretreatment with ZVAD‐FMK, a pan‐apoptosis inhibitor, completely prevented KC loss, whereas the necrosis inhibitor Necrostatin‐1 had no effect. Furthermore, the caspase‐9 inhibitor Z‐LEHD‐FMK, which selectively blocks the intrinsic apoptotic pathway,^[^ ^36^ ^]^ inhibited KC loss (Figure S2E,F, Supporting Information). Overall, these findings suggest that VSV‐WT infection induces massive KC death by activating the intrinsic apoptotic pathway.

The correlation between KC loss and limited therapeutic outcomes during VSV‐WT treatment suggested that KCs are indispensable for the virus‐mediated control of liver metastasis. To test this hypothesis, we administered diphtheria toxin (DT) into tumor‐bearing *Clec4f*‐iDTR mice concurrently with VSV‐M51R injection (Figure 2M). This led to the ablation of resident KCs after they had internalized the injected viral particles, thereby preventing potential morbidity and mortality associated with uncontrolled viral dissemination in the absence of KCs. Indeed, the antitumor effect of VSV‐M51R was completely abolished by KC depletion (Figure 2N,O; Figure S2G,H, Supporting Information). We next investigated whether preventing KC apoptosis could restore the therapeutic efficacy of VSV‐WT. For this purpose, we exploited our previously reported recombinant bacteria‐based BIL‐CRISPR approach^[^ ^15^ ^]^ to simultaneously disrupt the expression of Bak and Bax in KCs in vivo, which are core components that activate the intrinsic apoptotic pathway.^[^ ^36^ ^]^ As expected, disruption of Bak and Bax prevented KC loss and enhanced the antitumor effects of VSV‐WT treatment (Figure S2I–K, Supporting Information). Furthermore, we generated *Clec4f*‐cre: R26‐LSL‐*Bcl2* mice, in which the anti‐apoptotic protein Bcl2 was selectively overexpressed in KCs. Bcl2 overexpression markedly inhibited KC loss (Figure S2L,M, Supporting Information) and significantly improved the therapeutic efficacy of VSV‐WT (Figure S2N, Supporting Information). Collectively, these data highlight the critical role of KCs in virus‐mediated antitumor effects, and demonstrate that KC resistance to apoptosis dictates the therapeutic efficacy of VSV virotherapy against liver metastasis.

### 2.3 Peritumoral KCs Proliferate and take up Tumor Antigens Following VSV‐M51R Treatment

**Figure 3:**
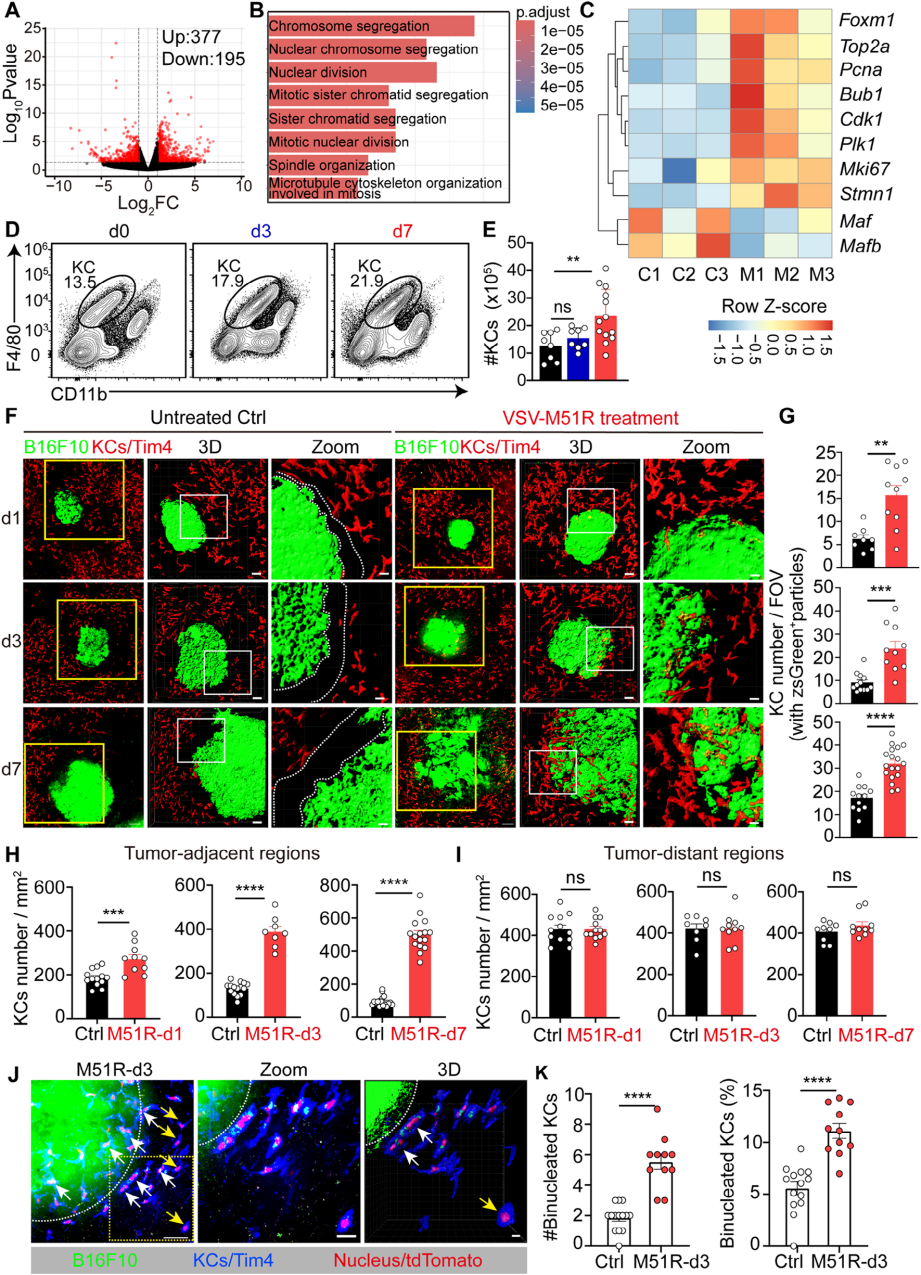
Peritumoral KCs proliferate and take up tumor antigens following VSV‐M51R treatment. A) Volcano plot showing DEGs between KCs from control and VSV‐M51R‐treated tumor‐bearing mice. B) GO functional enrichment analysis. C) Heatmaps of upregulated genes related to cell proliferation. C, untreated control; M, VSV‐M51R‐treated. D) Representative flow cytometric plot of KCs at the indicated time points after VSV‐M51R treatment. E) Quantification of the KC number in D. Data from 8–13 mice. F) KC localization in proximity to liver metastases at the indicated time points after control or VSV‐M51R treatment. G) Quantification of the number of KCs containing zsGreen^+^ particles per FOV in F. H) Quantification of KC density in the peritumoral region (0–200 µm away from the tumor margin) or I) distant tumor areas (600 µm away from the tumor margin) in F. J) Representative intravital images showing binucleated KCs in B16F10‐ZsGreen tumor‐bearing *Clec4f*‐ntdTomato mice on day 3 after VSV‐M51R treatment. Scale bars, left panel, 100 µm; middle panel, 10 µm; right panel, 5 µm. K) Quantification of the number (left) and ratio (right) of binucleated KCs in the tumor‐adjacent area. Data are represented as mean ± SEM. ^*^ *p* < 0.05; ^**^ *p* < 0.01; ^***^ *p* < 0.001; ns, no significance; unpaired Student's t test for **G**, **H**, **I**, and **K**; one‐way ANOVA with Tukey's test for **E**. Each dot of **G**, **H**, **I**, and **K** represents an FOV, with 8–18 FOVs randomly selected from 3–4 mice per group.

To explore the mechanisms by which VSV‐M51R induces KC‐dependent antitumor effects in liver metastases, we performed bulk RNA sequencing (RNA‐seq) on KCs isolated from metastatic B16F10 melanoma‐bearing mice that were either untreated or treated with VSV‐M51R for 3 days. By this time point, VSV‐M51R had been completely cleared from KCs, and any potential interference from the viral genome and acute antiviral responses had subsided, enabling a more accurate assessment of the impact of the virus on KCs. The RNA‐seq data revealed a total of 574 differentially expressed genes (DEGs) between KCs from untreated and VSV‐M51R‐treated tumor‐bearing mice, with 377 genes upregulated and 195 genes downregulated after VSV‐M51R treatment (**Figure** **3**A). Intriguingly, Gene Ontology (GO) analysis revealed that pathways related to DNA replication and cell division were most prominently enriched (Figure 3B). Notably, common proliferation marker genes,^[^ ^37^ ^]^ including *Mki67*, *Top2a*, *Stmn1*, *Cdk1*, *Pcna, Bub1*, *Plk1*, and *Foxm1*, were upregulated in KCs from VSV‐M51R‐treated mice (Figure 3C). Additionally, genes associated with cyclins, the minichromosome maintenance (MCM) complex and the cell division cycle (CDC) family—all of which are essential for cell cycle progression—were prominently upregulated (Figure S3A–C, Supporting Information). Moreover, the expression of the transcription factors Mafb (encoded by *mafb*) and c‐Maf (encoded by *maf*), which are known to suppress the proliferative potential of macrophages, including KCs,^[^ ^15^, ^38^ ^]^ was reduced (Figure 3C). These data strongly suggest that VSV‐M51R treatment induces active cell proliferation in KCs. In line with these findings, we observed an approximately twofold increase in the overall number of KCs from day 3 to day 7 after VSV‐M51R treatment (Figure 3D,E).

Our previous study revealed that disrupting c‐Maf and Mafb expression in KCs led to their preferential expansion in peritumoral areas.^[^ ^15^ ^]^ The downregulation of these two transcription factors suggests that KC proliferation in response to VSV‐M51R treatment may follow a similar spatial pattern. Indeed, in vivo imaging revealed a progressive increase in the abundance of peritumoral KCs from day 1 to day 7 post virotherapy (Figure 3F,H), whereas the KC density in tumor‐distant areas was unchanged (Figure 3I). Accordingly, using Clec4f‐ntdTomato mice in which tdTomato is specifically expressed in the nucleus of KCs, we observed an increased number of dividing KCs in tumor‐adjacent areas on day 3 after VSV‐M51R treatment (Figure 3J,K). Furthermore, while Tim‐4^+^ resident KCs in untreated mice barely infiltrated into tumor foci, the increased abundance of peritumoral KCs in VSV‐M51R‐treated mice led to increased tumor infiltration of these macrophages (Figure 3F). 3D reconstruction further revealed that tumor‐infiltrating KCs formed intimate interactions with liver metastases and actively took up material from ZsGreen‐tagged metastatic melanoma cells (Figure 3F,G). Taken together, these data suggest that VSV‐M51R treatment induces KC proliferation in peritumoral areas, likely enhancing their capacity to access tumor antigens.

### 2.4 VSV‐M51R Treatment Augments KC Cross‐Presentation of Tumor Antigens

**Figure 4:**
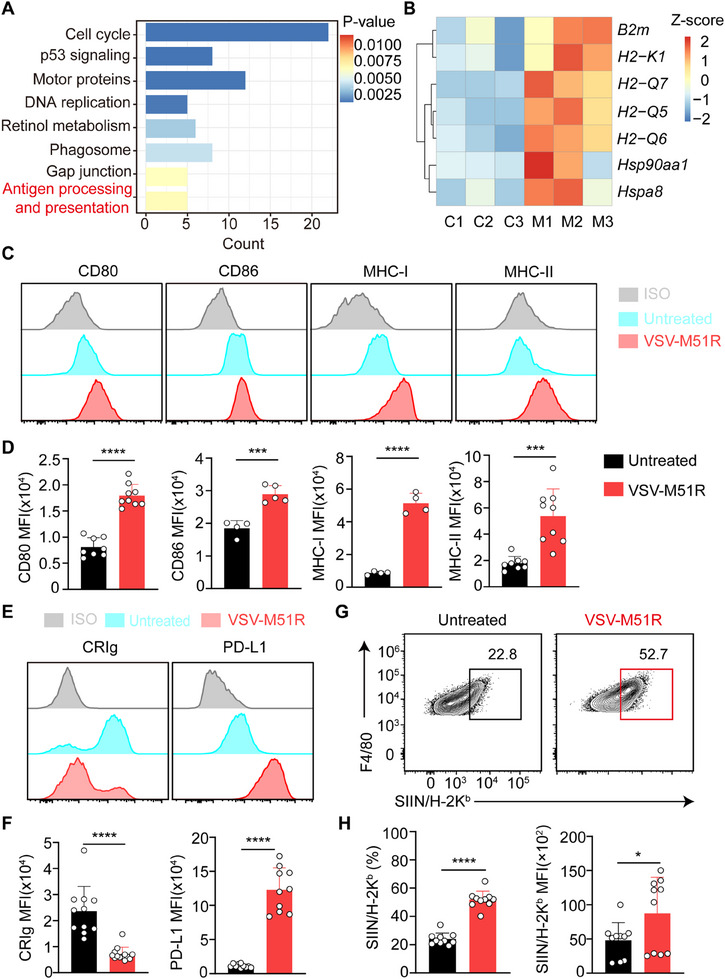
VSV‐M51R treatment augments KC cross‐presentation of tumor antigens. A) KEGG pathway enrichment analysis of DEGs between KCs from control and VSV‐M51R‐treated tumor‐bearing mice. B) Heatmaps of upregulated genes related to antigen processing and presentation. C, untreated control; M, VSV‐M51R‐treated. C) Representative histograms of CD80, CD86, MHC‐I and MHC‐II expression on KCs on day 3 post‐VSV‐M51R treatment. D) Mean fluorescence intensity (MFI) of CD80, CD86, MHCI and MHCII on KCs in C. Data from 4–9 mice per group. E) Representative histograms of CRIg and PD‐L1 expression on KCs on day 3 post‐VSV‐M51R treatment. F) MFI of CRIg and PD‐L1 on KCs was quantified. Data from 10–11 mice per group. G) Representative flow cytometric plot of SIINFEKL/H‐2K^b^ expression in KCs on day 3 following VSV‐M51R treatment. H) Quantification of the proportion and MFI of SIINFEKL/H‐2K^b^ in KCs. Data from 10 mice per group. Data are represented as mean ± SEM. ^*^ *p* < 0.05; ^**^ *p* < 0.01; ^***^ *p* < 0.001; ns, no significance (unpaired Student's t test).

Viral stimulation has the potential to subvert the tolerogenic properties of KCs, potentially enhancing their ability to cross‐prime hepatic CD8^+^T cell responses.^[^ ^18^, ^20^ ^]^ The increased acquisition of tumor‐derived materials by KCs following VSV‐M51R treatment prompted us to investigate whether their cross‐presentation capacity is also enhanced. Indeed, pathway analysis of our RNA‐seq data revealed enrichment of genes related to antigen processing and presentation in KCs from VSV‐M51R‐treated mice (**Figure** **4**A). Specifically, genes encoding MHC class I (MHC‐I) molecules such as *B2m*, *H2‐K1*, *H2‐Q5*, *H2‐Q6*, and *H2‐Q7*, as well as molecular chaperones involved in antigen processing, such as *Hspa8* and *Hsp90a*,^[^ ^39^, ^40^ ^]^ were significantly upregulated in KCs after VSV‐M51R treatment (Figure 4B). Consistently, the surface expression of MHC‐I, MHC‐II, and the costimulatory receptors CD80 and CD86 was elevated on KCs from VSV‐M51R‐treated mice compared with those from untreated tumor‐bearing mice (Figure 4C,D). We also examined the expression of coinhibitory molecules that can impair T‐cell priming by KCs. Notably, complement receptor of the immunoglobulin superfamily (CRIg), also known as VSIG4, a potent T‐cell‐inhibitory molecule highly and constitutively expressed on KCs,^[^ ^41^ ^]^ was significantly downregulated following VSV‐M51R treatment, with approximately two‐thirds of Tim‐4^+^ resident KCs completely losing CRIg expression (Figure 4E,F). However, programmed death 1 ligand 1 (PD‐L1) expression was elevated on KCs (Figure 4E,F), likely due to virus‐stimulated interferon responses, which are known to induce PD‐L1 expression.^[^ ^42^ ^]^ Additionally, the upregulation of MHC class I molecules, costimulatory receptors and molecular chaperones was similarly observed in primary human KCs stimulated with VSV‐M51R in vitro (Figure S3D–F, Supporting Information). Hence, these data strongly suggest that VSV‐M51R treatment may enhance the cross‐presentation capacity of KCs. To verify this, we established liver metastases in mice using B16F10 cells or MC38 cells expressing chicken ovalbumin (OVA) as a surrogate tumor antigen (B16F10‐OVA or MC38‐OVA) and assessed the KC expression of MHC‐I bound to SIINFEKL, a dominant CD8^+^ T‐cell epitope derived from OVA, at 3 days post‐VSV‐M51R treatment. The results revealed a significant increase in both the proportion and expression intensity of KCs presenting SIINFEKL/H‐2K^b^ following VSV‐M51R treatment (Figure 4G,H; Figure S3G,H, Supporting Information), confirming the augmented cross‐presentation of tumor antigens by KCs.

### 2.5 VSV‐M51R Treatment Elicits Robust KC‐Dependent Antitumor CD8^+^T Cell Responses

**Figure 5:**
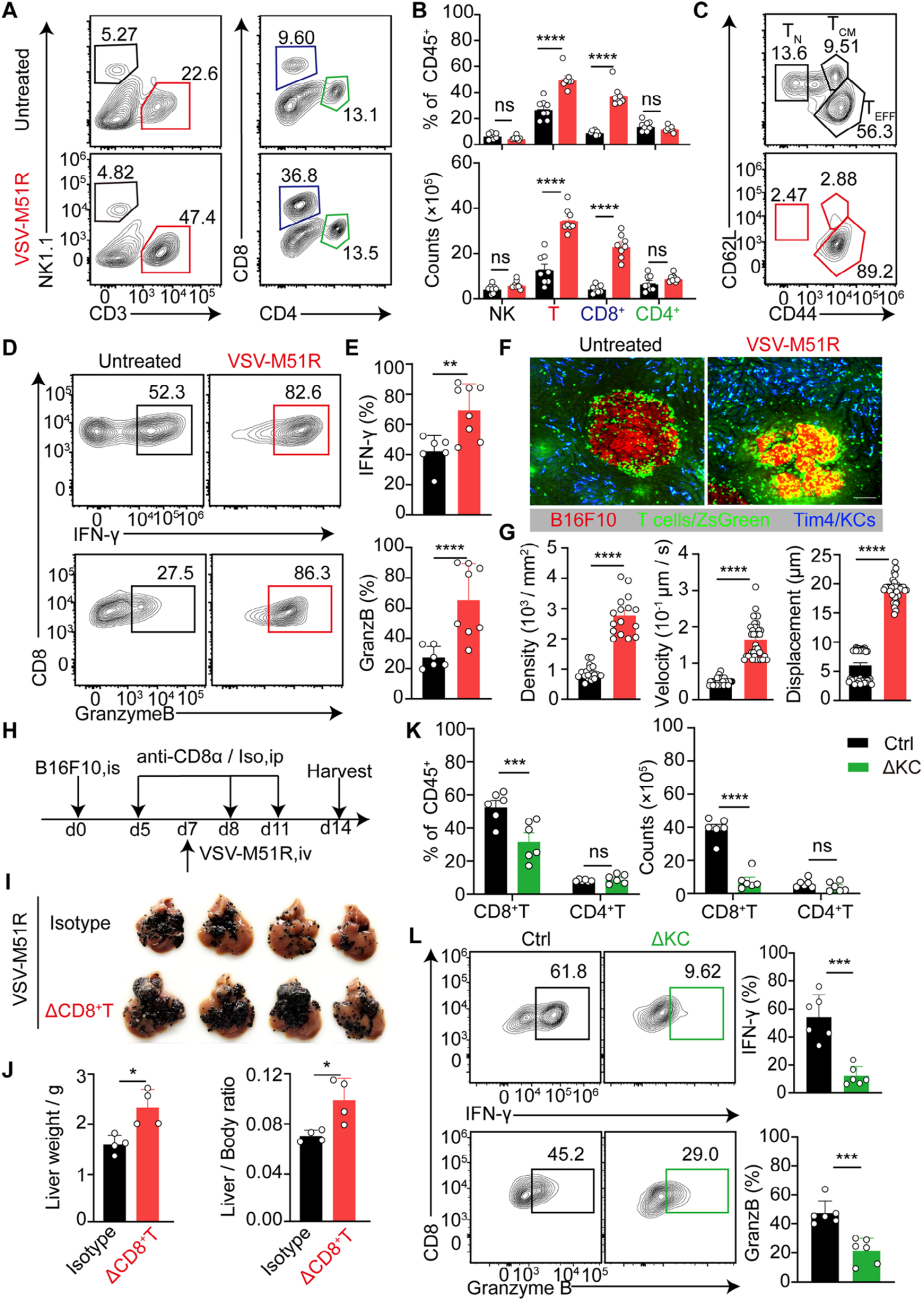
VSV‐M51R elicits robust KC‐dependent antitumor CD8^+^ T‐cell responses. A) Representative flow cytometric plots of hepatic NK, T, CD8^+^ T and CD4^+^ T cells at 7 days after VSV‐M51R treatment. B) Quantification of the proportions and numbers of NK, T, CD8^+^ T and CD4^+^ T cells. Data from 7–8 mice per group. C) Representative flow cytometric plots of T_N_, T_CM_, and T_EFF_ cells at 7 days post‐VSV‐M51R treatment, gated on hepatic CD8^+^T cells. D) Representative flow cytometric plots of IFN‐γ and Granzyme B expression by hepatic CD8^+^ T cells. E) Quantification of the ratio of IFN‐γ‐ or Granzyme B‐expressing CD8^+^ T cells in D. Data from 6–8 mice per group. F) Representative intravital images showing T‐cell infiltration in liver metastases at 7 days post‐VSV‐M51R treatment. G) The density, average velocity and displacement of intratumoral T cells were quantified. Each dot represents one tumor focus; n = 15–16 foci from 3 mice per group. H) Experimental design for I‐J. I) Liver photos were taken on day 7 after VSV‐M51R treatment with and without CD8^+^T cell depletion. J) Liver weight and the liver‐to‐body weight ratio were quantified. Data from 4 mice per group. K) Quantification of the proportions and numbers of hepatic CD8^+^ T and CD4^+^ T cells at 7 days post‐VSV‐M51R treatment in the presence or absence of KCs. Data from 6 mice per group. L) Representative flow cytometric plots and quantification of the ratio of IFN‐γ‐ or Granzyme B‐expressing CD8^+^ T cells. Data from 6 mice per group. Data are expressed as mean ± SEM. ^*^ *p* < 0.05; ^**^ *p* < 0.01; ^***^ *p* < 0.001; ns, no significance. Unpaired Student's t test for **E**, **G, J**, and **L**, and one‐way ANOVA with Tukey's test for **B** and **K**.

Given that VSV‐M51R treatment significantly increased the capacity of KCs to both take up and cross‐present tumor antigens, we hypothesized that this effect would lead to efficient induction of T‐cell responses against liver metastases. Profiling the composition of hepatic immune effector cells revealed a prominent increase in both the percentage and number of hepatic CD8^+^T cells on day 7 post‐VSV‐M51R treatment, whereas natural killer (NK) cells and CD4^+^T cells remained unchanged compared with those in the untreated group (**Figure** **5**A,B). Further analysis of the CD8^+^T cell population demonstrated an increase in the frequency of CD44^hi^CD62L^lo^ effector T cells (T_EFF_), accompanied by a proportional decrease in CD44^−^CD62L^hi^ naïve T cells (T_N_) and CD44^hi^CD62L^hi^ central memory T cells (T_CM_) among hepatic CD8^+^T cells, indicating a status of heighted activation (Figure 5C). Correspondingly, the ability of hepatic CD8^+^ T cells to secrete effector molecules, such as IFN‐γ and Granzyme B, was greater than that of the untreated group (Figure 5D,E). To assess the impact of virotherapy on tumor antigen‐specific CD8^+^T cell responses, we measured SIINFEKL/H‐2K^b^ tetramer^+^ CD8^+^T cells in the livers of B16F10‐OVA‐ or MC38‐OVA‐bearing mice and observed an increase in the percentage and effector function of these cells on day 3 post‐VSV‐M51R treatment (Figure S4A–H, Supporting Information). Moreover, endogenous antigen‐specific CD8^+^T responses against several reported neoepitopes in MC38 tumors, including Rpl18,^[^ ^43^ ^]^ Adpgk and Dpagt1,^[^ ^44^ ^]^ as measured by pMHC‐tetramer staining, were significantly increased in the livers of MC38 tumor‐bearing mice following VSV‐M51R treatment (Figure S4I,J, Supporting Information). Additionally, intravital imaging of tumor‐bearing *CD4‐cre*‐ZsGreen reporter mice, in which the hepatic ZsGreen^+^ cells were composed primarily of CD8^+^T cells after VSV‐M51R treatment (Figure S5A, Supporting Information), revealed enhanced T‐cell infiltration into the foci of liver metastases (Figure 5F,G). While T cells in untreated mice were mostly arrested at the tumor periphery, intratumoral T cells in VSV‐M51R‐treated mice exhibited increased motility (Figure 5G), a feature associated with active tumor immune surveillance by CD8^+^T cells.^[^ ^45^ ^]^ Taken together, these findings demonstrate that VSV‐M51R treatment provokes robust antitumor CD8^+^ T‐cell responses in liver metastases.

To validate the importance of CD8^+^ T‐cell responses in virotherapy‐induced tumor regression, we depleted T cells in tumor‐bearing *CD4‐cre*‐iDTR mice by administering DT prior to VSV‐M51R treatment. T‐cell depletion almost completely abrogated the therapeutic effect of VSV‐M51R (Figure S5B, Supporting Information). A similar loss of therapeutic efficacy was observed when CD8^+^T cells were selectively depleted with anti‐CD8α (Figure 5H–J), highlighting the indispensable contribution of CD8^+^ T cells to VSV‐M51R‐mediated tumor control. Considering the elevated IFN‐γ production by CD8^+^ T cells following VSV‐M51R treatment, we next investigated its contribution to the observed therapeutic effect. Although untreated IFN‐γ‐deficient (GKO) mice showed outgrowth of B16F10 metastases in the liver, reflecting an essential immune surveillance function of IFN‐γ in restricting the growth of liver metastases, VSV‐M51R treatment failed to reduce the tumor burden in these GKO mice (Figure S5C, Supporting Information). These findings suggest that CD8^+^ T cells may exert their tumoricidal activity at least in part through IFN‐γ production.

Given the enhanced cross‐presentation of tumor antigens by KCs, we sought to explore the contribution of these APCs to the VSV‐M51R‐mediated induction of antitumor CD8^+^ T‐cell responses. To address this, we established B16F10 liver metastases in *Clec4f*‐iDTR mice and concurrently depleted KCs upon VSV‐M51R treatment (Figure S5D, Supporting Information). Compared with those of control mice, the proportions and numbers of CD8^+^ T cells but not CD4^+^ T cells in the livers of KC‐depleted mice were significantly lower (Figure 5K, S5E). Moreover, the activation of hepatic CD8^+^ T cells (Figure S5F,G, Supporting Information), as well as their production of IFN‐γ and Granzyme B, was also strikingly diminished in the absence of KCs (Figure 5L). These findings confirm the indispensable role of KCs in eliciting efficient antitumor CD8^+^ T‐cell responses. In summary, VSV‐M51R treatment significantly enhances the ability of KCs to take up and cross‐present tumor antigens, thereby triggering robust antitumor T‐cell responses that effectively control liver metastases.

### 2.6 Mitochondrial Antiviral Signaling Protein (MAVS)‐Dependent IFN‐I Signaling Licenses KCs for Cross‐Presentation

**Figure 6:**
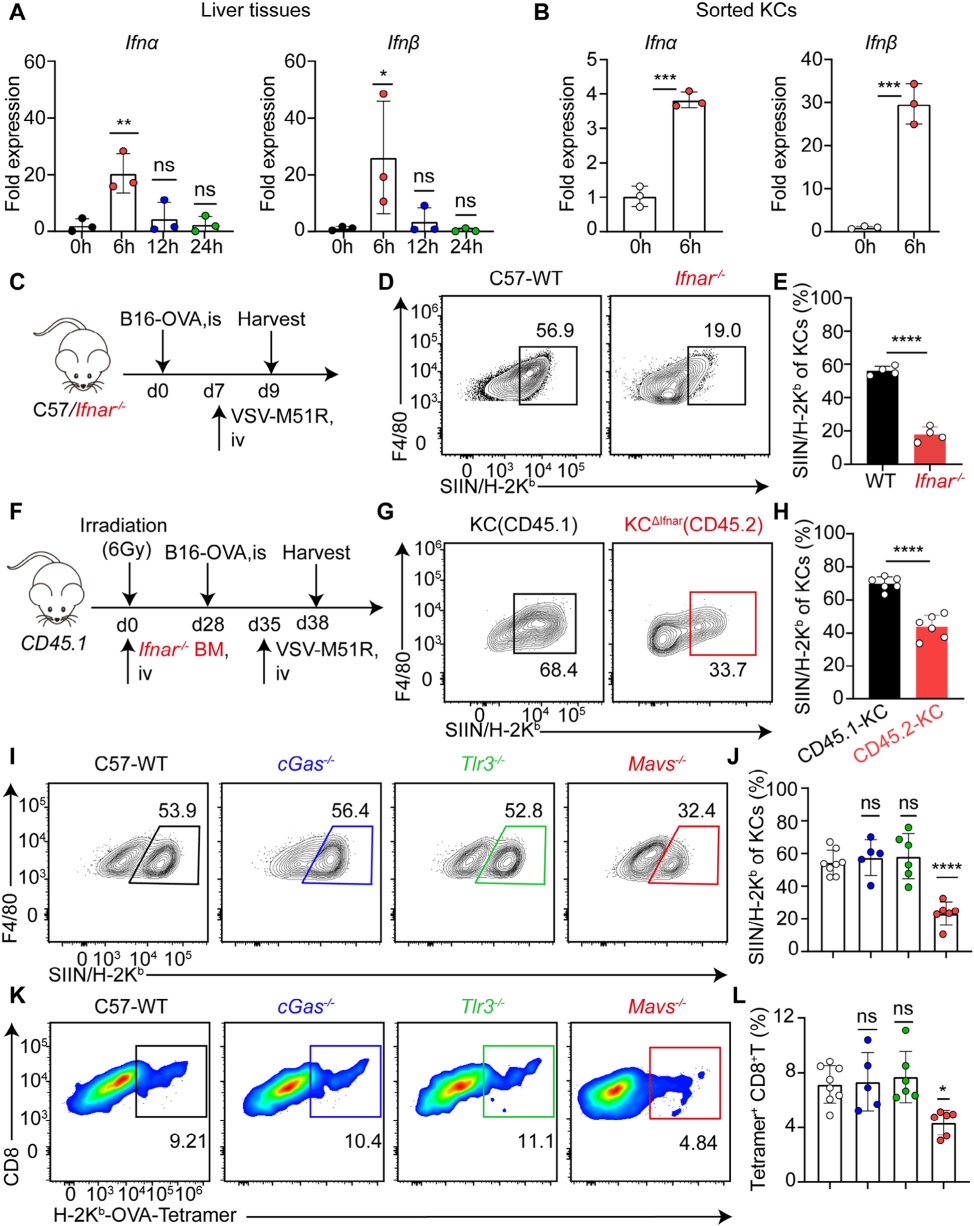
MAVS‐dependent IFN‐I signaling licenses KCs for cross‐presentation. A) mRNA expression levels of *Ifnα* and *Ifnβ* in liver tissues at the indicated time points after VSV‐M51R infection. B) mRNA levels of *Ifnα* and *Ifnβ* in KCs sorted from VSV‐M51R‐infected mice. Data from 3 mice per group. C) Liver metastasis of OVA‐expressing B16F10 melanoma was established in C57BL/6 or *Ifnar^−/−^* mice, followed by intravenous treatment with VSV‐M51R. D) Representative flow cytometric plots and E) quantification of SIINFEKL/H‐2K^b^ expression in KCs two days after VSV‐M51R treatment. Data from 4 mice per group. F) Experimental design for partial reconstitution of KCs. Sublethally irradiated CD45.1 mice were reconstituted with bone marrow cells from *Ifnar^−/−^* CD45.2 mice for 4 weeks, followed by the establishment of liver metastases and VSV‐M51R treatment. G) Representative flow cytometric plots and H) quantification of SIINFEKL/H‐2K^b^ expression on CD45.1 (WT) or CD45.2 (*Ifnar^−/−^*) KCs after VSV‐M51R treatment. Data from 6 mice per group. I) Representative flow cytometric plots and J) quantification of SIINFEKL/H‐2K^b^ expression in KCs from *cGas^−/−^*, *Tlr3^−/−^*, *Mavs^−/−^* and WT C57BL/6 mice 3 days after VSV‐M51R treatment. Data from 5–8 mice per group. K) Representative flow cytometric plots and L) quantification of SIINFEKL/H‐2K^b^ tetramer^+^ CD8^+^ T cells in the livers of *cGas^−/−^*, *Tlr3^−/−^*, *Mavs^−/−^* and WT C57BL/6 mice. Data from 5–8 mice per group. Data are represented as mean ± SEM. ^*^ *p* < 0.05; ^**^ *p* < 0.01; ^***^ *p* < 0.001; ns, no significance. Unpaired Student's *t* test for **B**, **E**, and **H** and one‐way ANOVA with Tukey's test for **A**, **J**, and **L**.

Type I interferon (IFN‐I) is a well‐known stimulator of T‐cell cross‐priming.^[^ ^46^ ^]^ KCs have been reported to be the primary early producers and responders of IFN‐I during systemic viral infections,^[^ ^47^, ^48^ ^]^ and we demonstrated that VSV‐M51R treatment strongly stimulated IFN‐I production in KCs (**Figure** **6**A,B), suggesting that the increased induction of KC cross‐presentation following virotherapy was likely dependent on IFN‐I signaling. To test this hypothesis, IFN‐I receptor‐deficient (*Ifnar^−/−^*) mice bearing metastatic B16F10‐OVA melanomas were systemically administered VSV‐M51R (Figure 6C). As expected, IFNAR deficiency resulted in significantly decreased expression of OVA peptide‐loaded MHC‐I on KCs, as measured by surface staining for the SIINFEKL/H‐2K^b^ pMHC complex, indicating impaired cross‐presentation of tumor antigens (Figure 6D,E). To further investigate whether IFN‐I directly acted on KCs, we generated partial bone marrow chimeras in which a proportion of CD45.1 resident KCs were replaced by their *Ifnar^−/−^* CD45.2 bone marrow‐derived counterparts (Figure 6F). These mice were treated with VSV‐M51R 7 days after intrasplenic inoculation of B16F10‐OVA tumors. Compared with IFNAR‐competent KCs, IFNAR‐deficient KCs presented much lower surface expression of SIINFEKL/H‐2K^b^ pMHC complexes (Figure 6G,H), suggesting that IFN‐I triggering of KCs is required for enhancing their cross‐presentation of tumor antigens.

To exclude the possibility that IFN‐I directly stimulates T cells instead of APCs during cross‐priming,^[^ ^49^ ^]^ we adoptively transferred WT or *Ifnar* ^−/−^ T cells into the lymphopenia *Rag1^−/−^* mice (Figure S6A, Supporting Information). T cells of both genotypes were successfully reconstituted in *Rag1^−/−^* recipients (Figure S6B, Supporting Information) and showed comparable responses following VSV‐M51R treatment of liver metastases (Figure S6C–G, Supporting Information), further suggesting that KCs, rather than T cells or B cells, were the primary targets of IFN‐I stimulation during virotherapy. Notably, we were unable to directly detect T‐cell responses in tumor‐bearing *Ifnar^−/−^* mice, as these mice rapidly succumbed to VSV‐M51R within three days after intravenous administration, possibly due to unrestrained viral infections. Multiple intracellular signaling pathways contribute to IFN‐I production during VSV infection in different cell types, such as the RIG‐I‐MAVS,^[^ ^50^ ^]^ cGAS‐STING^[^ ^51^ ^]^ and endosomal toll like receptor 3 (TLR3) pathways.^[^ ^52^ ^]^ We tested the involvement of each of these pathways in VSV‐M51R‐augmented cross‐priming by KCs. While KCs from *cGas^−/−^* and *Tlr3* ^−/−^ mice were able to efficiently cross‐present tumor antigens upon VSV‐M51R treatment, KCs from *Mavs* ^−/−^ mice were significantly impaired (Figure 6I,J). Accordingly, the induction of tumor antigen‐specific CD8^+^ T‐cell responses in the liver was largely diminished in *Mavs* ^−/−^ mice compared with that in *cGas^−/−^* or *Tlr3* ^−/−^ mice (Figure 6K,L). No significant morbidity or mortality was observed in any of these tumor‐bearing mouse strains upon VSV‐M51R treatment, suggesting that the blunted IFN‐I production was sufficient to protect against viral infection, although it compromised KC cross‐priming of hepatic antitumor T cells. These data collectively suggest that the MAVS‐IFN‐I signaling pathway is required to license KCs to efficiently cross‐prime hepatic antitumor CD8^+^ T cells during VSV‐M51R treatment of liver metastases.

### 2.7 PD‐L1 Blockade Potentiates the VSV‐M51R‐Mediated Antitumor Effect Against Advanced Liver Metastasis

Although we showed that a single intravenous infusion of VSV‐M51R at the early stage of liver metastasis was sufficient to induce tumor regression (Figure 1), the therapeutic effects of VSV‐M51R virotherapy on late‐stage liver metastasis, which represents a more clinically relevant scenario, remain unclear. To this end, we administered VSV‐M51R on day 12 after intrasplenic inoculation of B16F10 melanoma cells to mimic advanced liver metastasis, as untreated mice typically start reaching the experimental endpoint around day 15.^[^ ^15^ ^]^ However, the results indicated that a single infusion of VSV‐M51R had a minimal therapeutic effect on advanced liver metastases, demonstrating that VSV‐M51R virotherapy alone is insufficient to achieve tumor regression in late‐stage liver metastasis (Figure S7A, Supporting Information).

**Figure 7:**
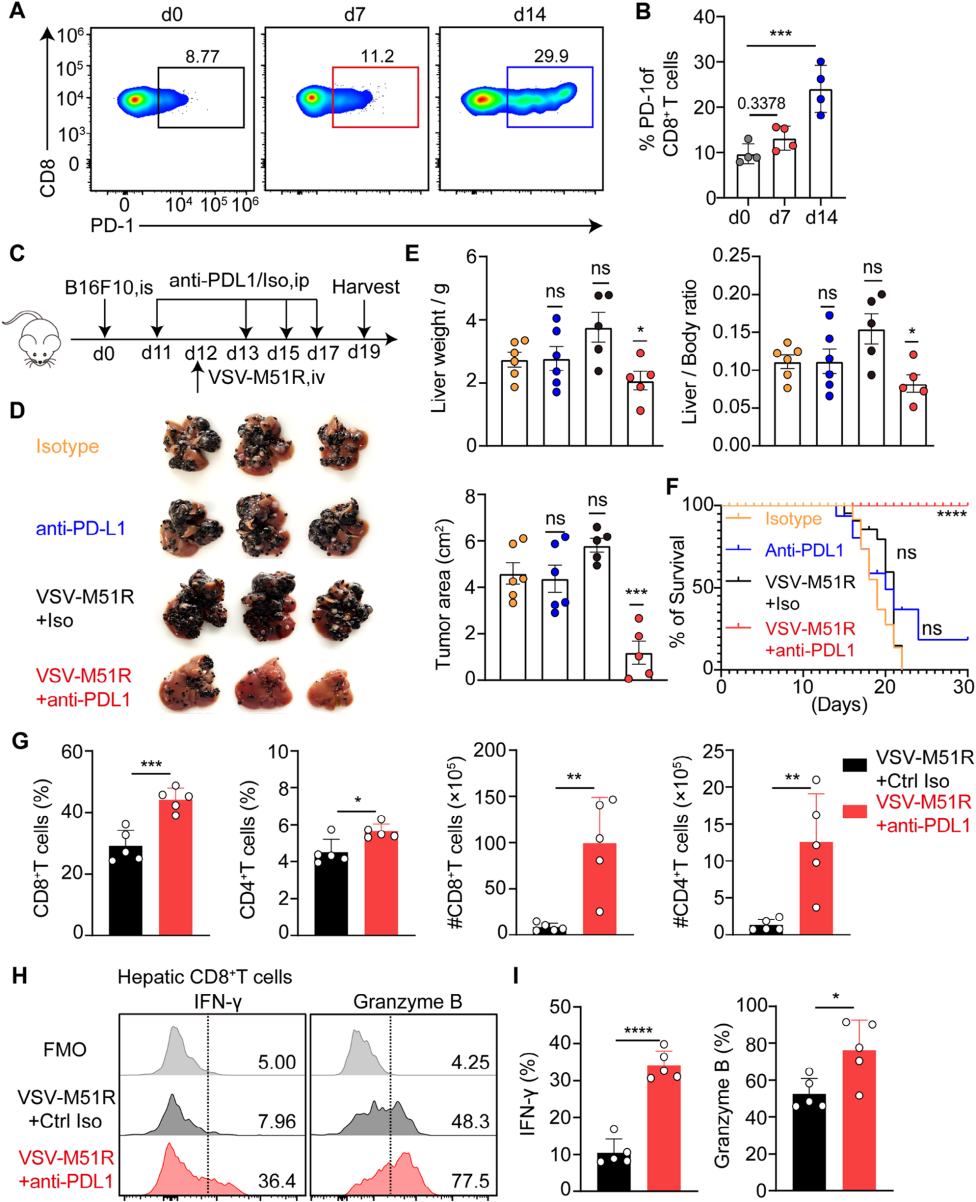
PD‐L1 blockade potentiates VSV‐M51R virotherapy against advanced liver metastasis. A) Representative flow cytometric plot and B) quantification of PD‐1 expression on hepatic CD8^+^ T cells during the progression of B16F10 liver metastases. Data from 4 mice per group. C) Experimental design for combinatory treatment of late‐stage liver metastasis using VSV‐M51R and anti‐PD‐L1 antibodies. D) Mice were treated with VSV‐M51R and anti‐PD‐L1 on day 12 following B16F10 tumor inoculation and were harvested on day 19 as illustrated. E) Liver weight, the ratio of liver weight to body weight and the tumor area were quantified. Data from 5–6 mice per group. F) Mouse survival rates were monitored. Pooled data from 10–15 mice per group. G) Quantification of the proportions and numbers of hepatic CD4^+^ and CD8^+^ T cells. Data from 5 mice per group. H) Representative histograms and I) quantification of IFN‐γ and Granzyme B expression in hepatic CD8^+^ T cells after combined VSV‐M51R and anti‐PD‐L1 treatment. Data from 5 mice per group. Data are expressed as mean ± SEM. ^*^ *p* < 0.05; ^**^ *p* < 0.01; ^***^ *p* < 0.001; ns, no significance. Unpaired Student's t test for **G** and **I**, one‐way ANOVA with Tukey's test for **B** and **E**. Two‐sided log‐rank test for **F**.

VSV‐M51R treatment induced a striking upregulation of PD‐L1 expression on KCs (Figure 4E,F). Given that PD‐1 expression gradually increases on hepatic CD8^+^T cells during the progression of liver metastasis (**Figure** **7**A,B), the intensified PD‐1/PD‐L1 interaction may impede the antitumor CD8^+^T cell responses triggered by virotherapy, particularly in late‐stage liver metastasis. Therefore, we combined VSV‐M51R with anti‐PD‐L1 to treat advanced liver metastasis. While PD‐L1 blockade alone had no therapeutic effect on liver metastases, which is consistent with the well‐documented resistance of liver metastasis to immune checkpoint inhibitors,^[^ ^3^, ^4^ ^]^ the combination treatment significantly reduced the hepatic metastatic tumor burden and prolonged the survival of tumor‐bearing mice compared to VSV‐M51R treatment alone, with a proportion of mice achieving near‐complete tumor eradication (Figure 7C–F; Figure S7B, Supporting Information). This enhanced therapeutic efficacy was accompanied by a greater than 10‐fold increase in hepatic CD8^+^ T cells, with a smaller increase in CD4^+^T cells (Figure 7G). Further analysis of hepatic CD8^+^ T cells revealed significantly increased production of effector molecules, such as IFN‐γ and Granzyme B (Figure 7H,I). Collectively, these results demonstrate that simultaneous blockade of PD‐L1 potentiates the therapeutic efficacy of VSV‐M51R virotherapy against advanced liver metastases by reinvigorating robust antitumor T‐cell responses.

## 3 Discussion

Systemic infusion of immunostimulatory recombinant virus holds promise for targeting and functionally revitalizing KCs in the treatment of liver malignancies. However, KCs are susceptible to death upon various viral infections, particularly when high viral doses are delivered intravenously to meet therapeutic requirements. Therefore, an appropriate viral platform capable of stimulating the antitumor function of KCs while retaining their integrity is crucial. Additionally, the potential risks of viral persistence and viral genome integration should be minimized to improve safety for clinical usage. In these regards, VSV‐M51R has emerged as an ideal candidate for developing KC‐targeted virotherapies. Recombinant VSV has demonstrated safety in clinical trials even with systemic administration.^[^ ^53^, ^54^ ^]^ The M51R mutation further reduces the potential toxicity of VSV by abrogating the virus's ability to inhibit host cell antiviral responses while simultaneously enhancing its immunostimulatory capacity.^[^ ^33^ ^]^ Moreover, VSV can be easily manipulated, allowing the engineering of genetic payloads into the virus. The self‐limited replication of VSV‐M51R within KCs could enable temporal and controllable expression of these therapeutic payloads, further increasing its therapeutic efficacy. Therefore, our findings highlight VSV‐M51R as a feasible, versatile and nontoxic viral platform for developing KC‐targeted immunotherapies against liver malignancies.

Although VSV‐WT relies primarily on the M protein to activate the intrinsic pathway of apoptosis in cancer cells,^[^ ^55^ ^]^ the VSV‐M51R mutant can alternatively trigger Fas‐ and Caspase‐8‐mediated extrinsic pathways of apoptosis in host cells.^[^ ^56^, ^57^ ^]^ Moreover, adenovirus induces massive KC necrosis following high‐dose intravenous injection.^[^ ^29^ ^]^ It remains unclear why KCs are resistant to VSV‐M51R‐induced cell death but are vulnerable to other viral infections; one possibility is that their intrinsic susceptibility to different types of cell death may vary. It is conceivable that other VSV mutants capable of both sparing KCs from death and enhancing their antitumor activity may also offer therapeutic benefits against multifocal liver metastasis. Understanding the underlying molecular mechanisms could not only guide the development of KC‐targeting viral platforms but also provide insights into KC fate determination, as the loss of resident KCs is frequently observed during liver inflammation and is associated with disease progression.^[^ ^58^ ^]^

It has been increasingly recognized that OVs can function beyond their direct cancer cell cytotoxicity by targeting noncancerous cells to elicit potent antitumor immunity.^[^ ^24^, ^59^, ^60^ ^]^ This is particularly true for intravenous OV delivery because of the widespread viral distribution. Recent studies have demonstrated that systemic administration of VSV‐∆M51 efficiently promotes tumor regression in infection‐resistant subcutaneous tumors, an effect that relies on the activation of conventional type 1 DCs (cDC1s) and their cross‐priming of antitumor T cells in tumor‐draining lymph nodes.^[^ ^24^, ^61^, ^62^ ^]^ These findings reinforce previous findings that VSV infection of myeloid cells in secondary lymphoid organs (SLOs) is critical for antiviral T‐cell priming.^[^ ^63^ ^]^ Although these studies reported no substantial VSV replication in KCs, our findings clearly indicate that VSV‐M51R can transiently infect and replicate within KCs early after intravenous administration. This discrepancy may be attributed to differences in viral strains, infectious dosages or observation time points. Importantly, we observed that VSV‐M51R elicited robust, KC‐dependent intrahepatic anti‐tumor CD8^+^T cell responses, suggesting that the mechanisms involved in T‐cell activation in liver metastases may differ from those in subcutaneous tumor models.

In addition to DCs, macrophages, including KCs, also possess the ability to cross‐present engulfed antigens.^[^ ^64^, ^65^ ^]^ In fact, KCs are fully capable of cross‐priming hepatic T‐cell responses during acute viral infections.^[^ ^20^ ^]^ It is thus temping to propose here that VSV‐M51R‐stimulated KCs interact with and cross‐present tumor antigens to naïve CD8^+^T cells recirculating through the bloodstream, leading to their activation and active surveillance of metastatic liver tumors. Although the potential involvement of DC‐mediated cross‐priming of antitumor T cells in SLOs cannot be ruled out, the fact that the spleen was removed in our liver metastasis model suggests a limited contribution of this process. In support of these findings, a recent study demonstrated that β‐glucan treatment stimulates antitumor T‐cell responses against pancreatic cancer liver metastasis by activating cross‐presentation in KCs, even with an intact spleen in this model.^[^ ^17^ ^]^ Moreover, while wild‐type VSV infection is sufficient to activate the cross‐priming function of DCs,^[^ ^63^, ^66^ ^]^ it barely retards the growth of liver metastases due to the induction of KC lysis, further underscoring the indispensable contribution of KC‐mediated intravascular cross‐priming during VSV virotherapy. Nevertheless, it is possible that VSV‐M51R treatment provokes the production of inflammatory cytokines by KCs, such as IL‐1, IFN‐I and TNFα, which are essential for the full maturation and activation of DCs.^[^ ^24^, ^67^ ^]^ Future investigations into the coordination between KCs and DCs in eliciting hepatic antitumor T‐cell immunity would be important for advancing the development of virotherapy for liver metastasis.

Local IFN‐I triggering of KCs has been demonstrated in various types of systemic viral infections and is essential for restricting viral replication and dissemination.^[^ ^28^, ^47^ ^]^ Here, we showed that direct sensing of IFN‐I by KCs can also augment their cross‐presentation of internalized antigens during VSV‐M51R virotherapy. Antigen cross‐presentation occurs via two distinct pathways, the cytosolic pathway and the vacuolar pathway. Although macrophages are generally thought to favor the vacuolar pathway for cross‐presentation,^[^ ^68^ ^]^ the upregulation of the molecular chaperones Hsp90 (encoded by *Hsp90aa1*) and Hsp70 (encoded by *Hsp8a*) supports the hypothesis that IFN‐I may stimulate the cytosolic pathway of antigen cross‐presentation in KCs. Hsp90 is well documented for pulling endosomal antigens out into the cytosol,^[^ ^40^, ^69^ ^]^ whereas Hsp70 facilitates the translocation of cytosolic antigens into the proteasome for degradation.^[^ ^70^ ^]^ Their coordinated action may enable KCs to undergo cytosolic processing of engulfed tumor‐derived antigens. In addition, IFN‐I can reduce phagosomal acidification and prolong the retention of engulfed antigens, thereby promoting the cytosolic pathway of cross‐presentation.^[^ ^46^, ^71^ ^]^ This action may be particularly important for KCs, which are likely the most highly phagocytic cells in the body. Alternatively, KCs may employ a process called cross‐dressing to acquire preformed pMHC complexes directly from tumor cells without undergoing antigen processing. This finding is supported by our previous findings showing that KCs can “nibble” live tumor cells,^[^ ^15^ ^]^ a process reminiscent of trogocytosis, which facilitates the transfer of the pMHC complex from tumor cells to phagocytes.^[^ ^72^ ^]^ A recent study suggested that Poly I:C can enhance cross‐dressing in DCs, possibly in an IFN‐I‐dependent manner.^[^ ^73^ ^]^ Whether and how cross‐dressing is involved in the KC‐mediated cross‐priming of antitumor T cells merits further investigation.

In addition to the marked upregulation of MHC‐I and costimulatory molecules, VSIG4, a well‐characterized coinhibitory molecule^[^ ^41^ ^]^ constitutively expressed on KCs, is significantly downregulated upon VSV‐M51R treatment. This downregulation is consistent with previous findings that inflammatory stimuli can inhibit VSIG4 expression^[^ ^41^ ^]^ or promote its shedding.^[^ ^74^ ^]^ VSIG4 has been shown to directly suppress T‐cell activation and proliferation by mitigating TCR signaling upon antigen engagement.^[^ ^75^ ^]^ Therefore, its downregulation could be a critical mechanism by which VSV‐M51R subverts the tolerogenic property of KCs and promotes effective T‐cell priming. In contrast, PD‐L1 expression is elevated on KCs during VSV‐M51R treatment. Given that PD‐1 expression progressively increases on T cells as liver metastases grow, this could explain why VSV‐M51R treatment is less effective in treating late‐stage liver metastasis and provide a rationale for combining VSV‐M51R with PD‐1/PDL1 blockade in the treatment of advanced liver metastasis. Although OVs, including adenovirus and herpes simplex virus (HSV), have been tested for liver metastasis treatment in clinical trials, their overall clinical efficacy remains limited.^[^ ^76^, ^77^, ^78^ ^]^ Specifically, intravenous injection of adenovirus, which has been reported to induce rapid death of KCs,^[^ ^29^ ^]^ has shown no clinical benefits in all tested patients, further highlighting the importance of KCs in effective virotherapy for liver metastasis.^[^ ^77^ ^]^ Taken together, our findings suggest that targeting KCs with a combination of intravenous VSV‐M51R administration and immune checkpoint inhibitors could represent a clinically applicable approach for treating patients with advanced liver metastasis.

## 4 Experimental Section

### Mice

*C57BL/6* mice were purchased from Nanjing GemPharmatech Co. (Nanjing, China). *Clec4f‐Cre‐tdTomato*, *iDTR*, *Cd4‐Cre*, *Rosa26‐LSL‐zsGreen*, *Rosa26‐LSL‐tdTomato*, *Ifnγ^−/−^*, *cGas^−/−^*, *Tlr3^−/−^*, *Mavs^−/−^*, and *Ifnar1^−/−^ ko* mice were originally obtained from the Jackson Laboratory. *Clec4f‐CreER* and *Rosa26‐LSL‐BCL2‐2A‐tdTomato* mice were generated by Shanghai Model Organisms Center Inc. (Shanghai, China). All the mice were maintained in a specific pathogen–free (SPF) facility at USTC. Animal experiments were conducted under the guidelines of the animal care committee of USTC with the approval number #USTCACUC192401034. Unless otherwise stated, 6‐ to 8‐week‐old mice of both sexes were used. Age‐ and sex‐matched littermates of the knock‐in or knockout mice were used as controls.

### Cell Lines and Viruses

B16F10, 293T, Pan02, and Vero cells were purchased from the China Center of Type Culture Collection (CCTCC). The MC38, MC38‐OVA and B16F10‐OVA cell lines were kindly provided by Dr. Zhigang Tian (USTC, Hefei) and Dr. Shu Zhu (USTC, Hefei). B16F10 cell lines stably expressing ZsGreen or tdTomato fluorescent proteins were generated via lentiviral transduction. The cell lines were cultured in RPMI 1640 (Biosharp) or Dulbecco's modified Eagle medium (DMEM, Vivacell) supplemented with 10% fetal calf serum (Biological Industries) and 1% penicillin/streptomycin (Biosharp). GFP‐encoding VSV‐WT and VSV‐M51R, both Indiana strains, were previously reported.^[^ ^52^ ^]^ For virus propagation, Vero cells were cultured in α‐MEM supplemented with 10% FBS until they reached 80% confluence. The cell monolayer was infected with VSV‐WT or VSV‐M51R at a MOI of 0.01. The supernatant containing the VSV particles was collected after 72 h, centrifuged at 3000 × g for 20 min, and passed through a 0.45 µm filter. Virus quantification was performed via the median tissue culture infectious dose (TCID_50_) assay as described previously.^[^ ^79^ ^]^ The mRNA copy number of the VSV N gene in the tissues was determined via quantitative PCR (qPCR). Briefly, total RNA was extracted from tumor and control liver tissues using the TRIzol reagent (Invitrogen), and transcribed into cDNA via the HiScript III qRT SuperMix Kit (Vazyme Biotech). The purified VSV‐N plasmid at a concentration of 50 ng µL^−1^ was serially diluted to serve as a template for generating the standard curve. SYBR green‐based real‐time qPCR amplification was conducted using the qTOWER^3^ system (Analytik Jena, Germany). The primers for VSV‐N were as follows: forward primer: 5′‐TGA‐TAG‐TAC‐CGG‐AGG‐ATT‐GAC‐GAC‐3′; reverse primer: 5′‐CCT‐TGC‐AGT‐GAC‐ATG‐ACT‐GCT‐CTT‐3′.

### Animal Models and In Vivo Treatment

An intrasplenic injection‐based multifocal liver metastasis model was established as previously described.^[^ ^15^ ^]^ Briefly, 6‐ to 8‐week‐old mice were anesthetized with isoflurane, mouse fur at the left upper flank was removed with a razor, the spleen was exposed via a small incision in the left flank, and tumor cells were injected into the spleen using a 30‐gauge needle, followed by splenectomy 5 min after injection. Unless otherwise stated, a total of 3 × 10^5^ B16F10 cells, 3 × 10^5^ B16F10‐ZsGreen cells, 3 × 10^5^ B16F10‐OVA cells, 5 × 10^5^ MC38 cells, 5 × 10^5^ MC38‐OVA cells or 1 × 10^6^ Pan02 cells were injected in 50 µL of PBS to establish metastatic liver tumors. For in vivo virotherapy of metastatic B16F10 and MC38 tumors, tumor‐bearing mice received intravenous (i.v.) administrations of VSV or VSV‐M51R at a dose of 3 × 10^8^ PFU on day 7 post‐tumor inoculation. Alternatively, tumor‐bearing mice were i.v. injected with virus on day 12 to mimic late‐stage liver metastasis and were harvested on day 19. For the slow‐growing Pan02 metastatic tumors, tumor‐bearing mice were treated with VSV‐M51R on day 10 post‐tumor inoculation and harvested on day 21. Mice were monitored daily during this period, and an increase in body weight greater than 20% was considered to have reached the end point of the experiment. For combinatory treatment with anti‐PD‐L1 antibodies, mice were additionally injected intraperitoneally (i.p.) with 100 µg of anti‐PDL1 (#124 339, Biolegend) at days 11, 13, 15, and 17 after tumor inoculation as depicted.

To deplete KCs, *Clec4f*‐iDTR mice were i.p. injected with 200 ng DT (D0564, Millipore Sigma). To deplete T cells, *Cd4*‐iDTR mice were i.p. injected with 100 ng DT daily for 3 consecutive days. To deplete CD8^+^ T cells, tumor‐bearing mice were i.p. injected with 100 µg of anti‐CD8α (BE0061, Bio X Cell) antibody three times at days 5, 8, and 11 after tumor inoculation. For the fate‐mapping experiments, a dose of 1 mg tamoxifen (Millipore) dissolved in corn oil (Millipore) was orally administered to *Clec4f*‐CreERT2:R26‐ tdTomato mice three days before VSV‐M51R injection. For in vivo inhibition of cell death, the pan‐caspase inhibitor Z‐VAD‐FMK (10 mg kg^−1^), the caspase‐9 inhibitor Z‐LEHD‐FMK (6 mg kg^−1^) or the necroptosis inhibitor Necrostatin‐1 (5 mg kg^−1^) were dissolved and diluted in 200 µL of saline solution and were i.p. injected 30 min before VSV infection.

### Intravital Microscopy (IVM)

Surgical preparation of mice for IVM of the liver was conducted as previously described.^[^ ^15^ ^]^ Briefly, the tail vein of anesthetized mice was cannulated with PE‐10 tubing filled with sterile saline, allowing for the delivery of fluorescence‐conjugated antibodies and additional anesthetics. The following antibodies or dyes were used: Annexin V (1.4 µg), anti‐F4/80 (1.8 µg), anti‐TIM4 (2 µg), and PI (5 µg). Midline laparotomy was performed to exteriorize the left lateral lobe of the liver, which was then laid against the glass coverslip embedded in a customized temperature‐controllable sample holder. The surface of the liver was covered with strips of saline‐moistened Kimwipes (Fisher Scientific) to restrict movement. The sample holder fit the motorized microscope stage and was maintained at 37 °C during the course of the experiments. For spinning‐disk confocal IVM, example images were acquired via a Nikon Ti2‐E inverted microscope coupled with a Yogokawa CSU‐W1 spinning disk scanner. The microscope was equipped with four lasers (405, 488, 561, and 640 nm; Toptica) and four emission bandpass filters (447/60, 525/50, 617/73, and 685/40) to offer multichannel high‐resolution imaging that was recorded using an sCMOS camera (Prime95B, Photometrics). For two‐photon IVM, a Nikon AX R MP upright microscope system equipped with a resonance laser scanner head, a dual‐output pulsed laser (1040 nm and 660–1320 nm, Coherent Discovery) and GaAsP HyD detectors was used. Example images were analyzed and quantified via NIS‐Elements AR software (version 5.20.00) and ImageJ (Fiji). 3D reconstruction was conducted using Imaris (version 7.0, Bitplane).

### Cell Isolation and Flow Cytometry

Mouse livers were cut into small pieces and incubated with a digestion mixture comprising 10 mL of prewarmed DMEM supplemented with 0.5 mg mL^−1^ collagenase I (C0130, Millipore Sigma) and 5 U mL^−1^ DNase I (11 284 932 001, Millipore Sigma). Liver tissues were digested at 37 °C with shaking at 200 rpm (Constant temperature incubator shaker, #ZWY240, ZhiCheng) for 20 min and then filtered through a 70‐µm cell strainer (Biosharp). After the removal of hepatocytes and tissue debris by a short centrifugation, the cells were pelleted and washed by centrifugation at 400 × g at 4 °C for 5 min. Liver nonparenchymal cells (LNPCs) were then obtained following the lysis of red blood cells and were resuspended in ice‐cold 1× PBS. For surface marker staining, the cells (1 × 10⁶ in 100 µL) were incubated with an Fc blocker (2.4G2, Bio X Cell) for 20 min before being stained with a mixture of fluorophore‐conjugated antibodies for 30 min at 4 °C in the dark. For tetramer staining, the cells were stained with PE or Alexa 647‐labeled H‐2K^b^‐SIINFEKL tetramer (NIH) for 30 min at 4 °C in the dark prior to surface marker staining. For intracellular staining, the cells were pretreated with the Cell Activation Cocktail (with Brefeldin A) (423 304, BioLegend) for 4 h prior to surface marker staining, followed by fixation and permeabilization using the Foxp3 Staining Buffer Set (00‐5523‐00, eBioscience). DAPI (Biosharp) or Zombie Violet dyes (423 102, BioLegend) were used to exclude dead cells. Cell‐counting beads (424 902, BioLegend) were added to the samples before flow cytometric detection for cell enumeration. Data acquisition was carried out on either the BD Fortessa or Beckman CytoFLEX flow cytometer. Flow cytometric data were analyzed using the FlowJo (version 10.4). The following gating strategy was used to define the cell populations: KCs (CD45^+^ Ly6G^−^ CD11b^low^ F4/80^+^), NK cells (CD45^+^ NK1.1^+^), T cells (CD45^+^NK1.1^−^CD3^+^), CD8^+^ T cells (CD45^+^NK1.1^−^CD3^+^ CD8^+^) and CD4^+^ T cells (CD45^+^NK1.1^−^ CD3^+^ CD4^+^). The antibodies used for flow cytometry were as follows: FITC anti‐mouse/human CD11b (M1/70, Biolegend), FITC anti‐mouse CD3 (145‐2C11, Biolegend), FITC anti‐mouse CD62L (MEL‐14, Biolegend), FITC anti‐mouse CD80 (Armenian Hamster IgG, Biolegend), Alexa Fluor 488 anti‐mouse VSIG4 (NLA14, Biolegend), Alexa Fluor 488 anti‐mouse F4/80 (BM8, Biolegend), PE anti‐mouse Tim4 (RMT4‐54, Biolegend), PE anti‐mouse CD4 (RM4‐5, Biolegend), PE anti‐mouse IFN‐γ (XMG1.2, Biolegend), PE anti‐mouse H‐2K^b^ bound to the SIINFEKKL Antibody (25‐D1.16, Biolegend), PE anti‐mouse CD274 (10F.9G2, Biolegend), PE anti‐mouse CD86 (A17199A, Biolegend), PerCP/Cy5.5 anti‐mouse CD8a (53‐6.7, Biolegend), PerCP/Cy5.5 anti‐mouse H‐2K^b^ (AF6‐88.5, Biolegend), PE/Cyanine7 anti‐mouse Tim‐4 (RMT4‐54, Biolegend), PE/Cyanine7 anti‐mouse/human CD11b (M1/70, Biolegend), PE/Cyanine7 anti‐mouse CD45.1 (A20, Biolegend), PE/Cyanine7 anti‐mouse NK1.1 (PK136, Biolegend), APC anti‐mouse F4/80 (BM8, Biolegend), APC anti‐mouse CD44 (IM7, Biolegend), APC anti‐human/mouse Granzyme B Recombinant Antibody (QA16A02, Biolegend), APC anti‐mouse CD3 (17A2, Biolegend), Alexa Fluor 647 anti‐mouse Tim‐4 (RMT4‐54, Biolegend), APC/Cyanine7 anti‐mouse CD45 (30‐F11, Biolegend), APC/Cyanine7 anti‐mouse CD45.2 (104, Biolegend), APC/Cyanine7 anti‐mouse CD3ε (145‐2C11, Biolegend), Alexa Fluor 700 anti‐mouse Ly‐6G (1A8, Biolegend), Alexa Fluor 700 anti‐mouse CD45 (30‐F11, Biolegend), Brilliant Violet 421 anti‐mouse F4/80 (BM8, Biolegend), Brilliant Violet 421 anti‐mouse CD62L(MEL‐14, Biolegend), Brilliant Violet 605 anti‐mouse F4/80 (BM8, Biolegend), Brilliant Violet 510 anti‐mouse CD4 (RM4‐4, Biolegend), Brilliant Violet 510 anti‐mouse I‐A/I‐E (M5/114.15.2, Biolegend), Brilliant Violet 785 anti‐mouse/human CD11b (M1/70, Biolegend), Anti‐Calreticulin antibody (ab2907, Abcam), Donkey Anti‐Rabbit IgG H&L (Alexa Fluor 647, Abcam), APC anti‐human CD68 (Y1/82A, Biolegend), PE/Cyanine7 anti‐human CD45 (HI30, Biolegend), PE Rat IgG1, κ Isotype Ctrl (RTK2071, Biolegend), PE Mouse IgG1, κ Isotype Ctrl (MOPC21, Biolegend), FITC Rat IgG2a, κ Isotype Ctrl (RTK2758, Biolegend). H‐2K^b^ chicken ova 257–264 SIINFEKL PE‐labeled tetramer (NIH), H‐2K^b^ chicken ova 257–264 SIINFEKL Alexa 647‐labeled tetramer (NIH). Customized PE‐labeled H2K^b^‐RPL18 neoepitope KILTFDRL tetramer, H2D^b^‐Adpgk neoepitope ASMTNMELM tetramer and H2K^b^‐Dapgt1 neoepitope SIIVFNLL tetramer were obtained from the Gene‐Bio Ltd. (Hefei)

### Human KC Isolation and Treatment

Human liver tissues were obtained from donor liver grafts procured from brain‐dead individuals who met the Chinese Classification Criteria for organ donation after Cardiac Death. Informed consent for the use of donor organs in transplantation and scientific research was obtained from the donors’ next of kin. All procedures were critically reviewed and approved by the institutional ethics board of the First Affiliated Hospital of USTC (#AF/SC‐12‐2/04.0‐2024KY‐510). For KC isolation, liver tissue was cut into small pieces (1–2 mm^3^) and digested in prewarmed DMEM containing 2 mg mL^−1^ type IV collagenase (C5138, Millipore Sigma) at 37 °C with agitation at 100 rpm for 30 min (Constant temperature incubator shaker, #ZWY240, ZhiCheng). The samples were vortexed every 10 min during digestion. The resulting mixture was filtered through a 100‐µm cell strainer (Biosharp). The cell suspension was then centrifuged at 50 × g for 3 min at 4 °C to separate hepatocytes (pellets) from non‐parenchymal cells. The supernatant was collected and further centrifuged at 500 × g for 5 min at 4 °C. The resulting cell pellets were resuspended in 10 mL of DMEM and centrifuged again at 500 × g for 5 min at 4 °C. The final cell pellets were cultured in DMEM supplemented with 10% fetal calf serum and 1% penicillin/streptomycin. After a 4‐h incubation at 37 °C with 5% CO_2_, non‐adherent cells and debris were removed by gently washing with PBS. The adherent cells, which were composed predominantly of KCs, were maintained in DMEM supplemented with 10% fetal calf serum and 1% penicillin/streptomycin. The KC identity of these adherent cells was confirmed via flow cytometry. For VSV‐M51R treatment, KCs were seeded into 6‐well plates at a density of 5 × 10^5^ cells/well. After 12 h of culture to allow cell monolayer formation, the cells were infected with VSV‐M51R at a MOI of 0.6 and incubated for an additional 24 h. The cells were then harvested and subjected to qPCR analysis of the following molecules, using the following primers: *CD80* sense (5′‐CTC‐TTG‐GTG‐CTG‐GCT‐GGT‐CTT‐T‐3′) and *CD80* anti‐sense (5′‐GCC‐AGT‐AGA‐TGC‐GAG‐TTT‐GTG‐C‐3′); *CD86* sense (5′‐ CCA‐TCA‐GCT‐TGT‐CTG‐TTT‐CAT‐TCC‐3′) and *CD86* anti‐sense (5′‐ GCT‐GTA‐ATC‐CAA‐GGA‐ATG‐TGG‐TC‐3′); HLA‐A sense (5′‐ AGA‐TAC‐ACC‐TGC‐CAT‐GTG‐CAG‐C‐3′) and *HLA‐A* anti‐sense (5′‐ GAT‐CAC‐AGC‐TCC‐AAG‐GAG‐AAC‐C‐3′); *HLA‐B* sense (5′‐ CTG‐CTG‐TGA‐TGT‐GTA‐GGA‐GGA‐AG‐3′) and *HLA‐B* anti‐sense (5′‐ GCT‐GTG‐AGA‐GAC‐ACA‐TCA‐GAG‐C‐3′); *HLA‐C* sense (5′‐ GGA‐GAC‐ACA‐GAA‐GTA‐CAA‐GCG‐C‐3′) and *HLA‐C* anti‐sense (5′‐ ACA‐TCC‐TCT‐GGA‐GGG‐TGT‐GAG‐A‐3′); *HSP90AA1* sense (5′‐ TCT‐GCC‐TCT‐GGT‐GAT‐GAG‐ATG‐G‐3′) and *HSP90AA1* anti‐sense (5′‐ CGT‐TCC‐ACA‐AAG‐GCT‐GAG‐TTA‐GC‐3′); *HSPA8* sense (5′‐ TCC‐TAC‐CAA‐GCA‐GAC‐ACA‐GAC‐C‐3′) and *HSPA8* anti‐sense (5′‐ CAG‐GAG‐GTA‐TGC‐CTG‐TGA‐GTT‐C‐3′); *GAPDH* sense (5′‐ GTC‐AAG‐GCT‐GAG‐AAC‐GGG‐AA‐3′) and *GAPDH* anti‐sense (5′‐ AAA‐TGA‐GCC‐CCA‐GCC‐TTC‐TC‐3′);

### Bulk RNA Sequencing

KCs were isolated via a two‐step in situ perfusion procedure. Briefly, the mice were anesthetized with isoflurane, and a 26G catheter connected to the pump was inserted into the portal vein. The inferior vena cava was cut immediately after catheter insertion. The liver was perfused via the portal vein with an initial 20 mL of HBSS (with 0.5 mm EGTA) followed by 20 mL of perfusion buffer (HBSS containing calcium and magnesium + 0.05% collagenase IV) at a constant flow rate of 5 mL min^−1^. The entire liver was removed, minced and passed through a 70 µm cell strainer. The cell suspension was centrifuged at 50 × g for 3 min to remove liver parenchymal cells. The supernatant was then centrifuged at 400 × g for 5 min at 4 °C, and the resulting pellet was resuspended in 1 mL RBC lysis buffer for 1 min. LNPC was obtained after washing and was subsequently resuspended in 1 mL of HBSS buffer for antibody staining. Zombie^−^CD45^+^TIM4^+^KCs were sorted using a BD FACSAria III with a purity greater than 95%. A total of 20 000 to 50 000 cells were collected, and total RNA was extracted using the RNeasy Micro Kit (QIAGEN, USA). RNA quality was assessed via an Agilent Bioanalyzer (Agilent, USA), with RNA integrity numbers (RINs) ranging from 6–9. cDNA libraries were prepared from 2 ng of total RNA using the Smart‐Seq v2 protocol as previously reported.^[^ ^80^ ^]^ In brief, 50 pg of cDNA was processed with the Nextera XT DNA Library Preparation Kit (Illumina, USA) via 12–14 PCR cycles according to the manufacturer's instructions. The length distribution of the cDNA libraries was monitored using an Agilent Bioanalyzer. cDNA libraries that passed quality control (QC) were subjected to Illumina sequencing. For analysis of the transcriptomic data, poor‐quality reads were first filtered via Trimmomatic. The raw reads were aligned to the mouse reference genome GRCm38_M13 provided by GENCODE using STAR. The FeatureCounts tool was used to assign exonic reads to the corresponding genes. The expression data were imported into the R statistical environment (R version 4.1.1) for further analysis of differential gene expression. GO and KEGG pathway analyses were conducted with the “clusterProfiler” package in R.

### In Vivo KC Editing

In vivo editing of KCs was performed using the BIL‐CRISPR approach as we described previously.^[^ ^15^ ^]^ Briefly, two pairs of sgRNA oligos targeting different exons of *Bax* or *Bak* were designed and synthesized as follows: Bax‐exon1: GAC‐GGG‐TCC‐GGG‐GAG‐CAG‐CT; Bax‐exon2: GCT‐CTG‐AAC‐AGA‐TCA‐TGA‐AG; Bak‐exon1: CCG‐AAG‐GTG‐GGC‐TGC‐GAT‐GA; Bak‐exon2: ACC‐CCG‐AGA‐TGG‐ACA‐ACT‐TG. sgRNA oligos were then annealed and ligated sequentially into the pX459‐2U6‐BsaI‐SapI vectors using the BsaI‐ and SapI‐restricted enzymes. A total of 5–10 µg of sequence‐verified plasmids were electroporated into ClearColi competent *E. coli* (Lucigen). The transformed bacteria were plated onto LB agar media in 15 cm petri dishes in the presence of ampicillin (100 µg mL^−1^). Bacteria were gently scraped from the plates after 16–18 h of overnight culture, collected into a 50 mL centrifuge tube, weighed, and washed with saline. The bacterial number was calculated as follows: total CFU = 30 × bacterial weight (g) ×10^10^. For KC editing, 8‐week‐old mice were i.v. administered 10^9^ CFU ClearColi containing *Bax*/*Bak* dual editing plasmids or backbone vectors and waited for 7 days before further experiments.

### Statistical Analysis

Every experiment was independently repeated at least twice, with each group containing a minimum of three biological replicates. Statistical analysis was conducted using the GraphPad Prism software (version 9.0). Data were expressed as the mean ± SEM. Unpaired Student's *t* test was used for comparisons between 2 individual groups. One‐way ANOVA with Tukey's test was used for multiple group comparisons. Mouse survival was analyzed via a 2‐sided log‐rank test. All experiments were repeated at least twice, with 3–5 biological samples per group. ^*^ *p* < 0.05; ^**^ *p* < 0.01; ^***^ *p* < 0.001; ^****^ *p* < 0.0001; ns, no significance.

## Conflict of Interest

The authors declare no conflict of interest.

## Author Contributions

C.C. designed and conducted the majority of the experiments and analyzed the data. Q.Z., J.L., and X.Z. assisted in some experiments; J.W., D.G., and L.L. provided valuable experimental resources, critical thinking and technical assistance; Z.Z. conceived and supervised the study; C.C. and Z.Z. wrote the manuscript.

## Supporting information



Supporting Information

## Data Availability

The data that support the findings of this study are available from the corresponding author upon reasonable request.
