# Supplementary material for: Viral‐Directed Augmentation of Kupffer Cell Cross‐Presentation Provokes Antitumor Immunity Against Liver Metastasis
Source: Adv Sci (Weinh). 2025 Jul 29;12(39):e04929. doi: 10.1002/advs.202504929 (PMC12533311; doi:10.1002/advs.202504929)
Supplement: Supplementary file 1 — Supporting Information [file ADVS-12-e04929-s001.pdf]

ADVANCED SCIENCE
Open Access

Supporting Information

for *Adv. Sci.*, DOI 10.1002/advs.202504929

Viral-Directed Augmentation of Kupffer Cell Cross-Presentation Provokes Antitumor Immunity Against Liver Metastasis

Chen Chen, Qing Zhang, Jiajia Li, Xia Zhou, Daxing Gao, Lu Li, Dabing Huang, Jizhou Wang* and Zhutian Zeng**

Supplementary Materials for

Viral-directed augmentation of Kupffer cell cross-presentation provokes antitumor immunity against liver metastasis

Chen Chen[1], Qing Zhang[1], Jiajia Li[1], Xia Zhou[1], Daxing Gao[1], Lu Li[1], Dabing Huang[1#], Jizhou Wang[2#], Zhutian Zeng[1#]

1. State Key Laboratory of immune response and immunotherapy, Department of Oncology, The First Affiliated Hospital of USTC, Center for Advanced Interdisciplinary Science and Biomedicine of IHM, Division of Life Sciences and Medicine, University of Science and Technology of China, Hefei, Anhui, 230001, China.

2. Department of Hepatobiliary Surgery, Center for Leading Medicine and Advanced Technologies of IHM, Division of Life Sciences and Medicine, University of Science and Technology of China, Hefei, Anhui 230001, China.

# Correspondence should be addressed to hdabing@ustc.edu.cn, wangjoe@ustc.edu.cn or zengzt@ustc.edu.cn

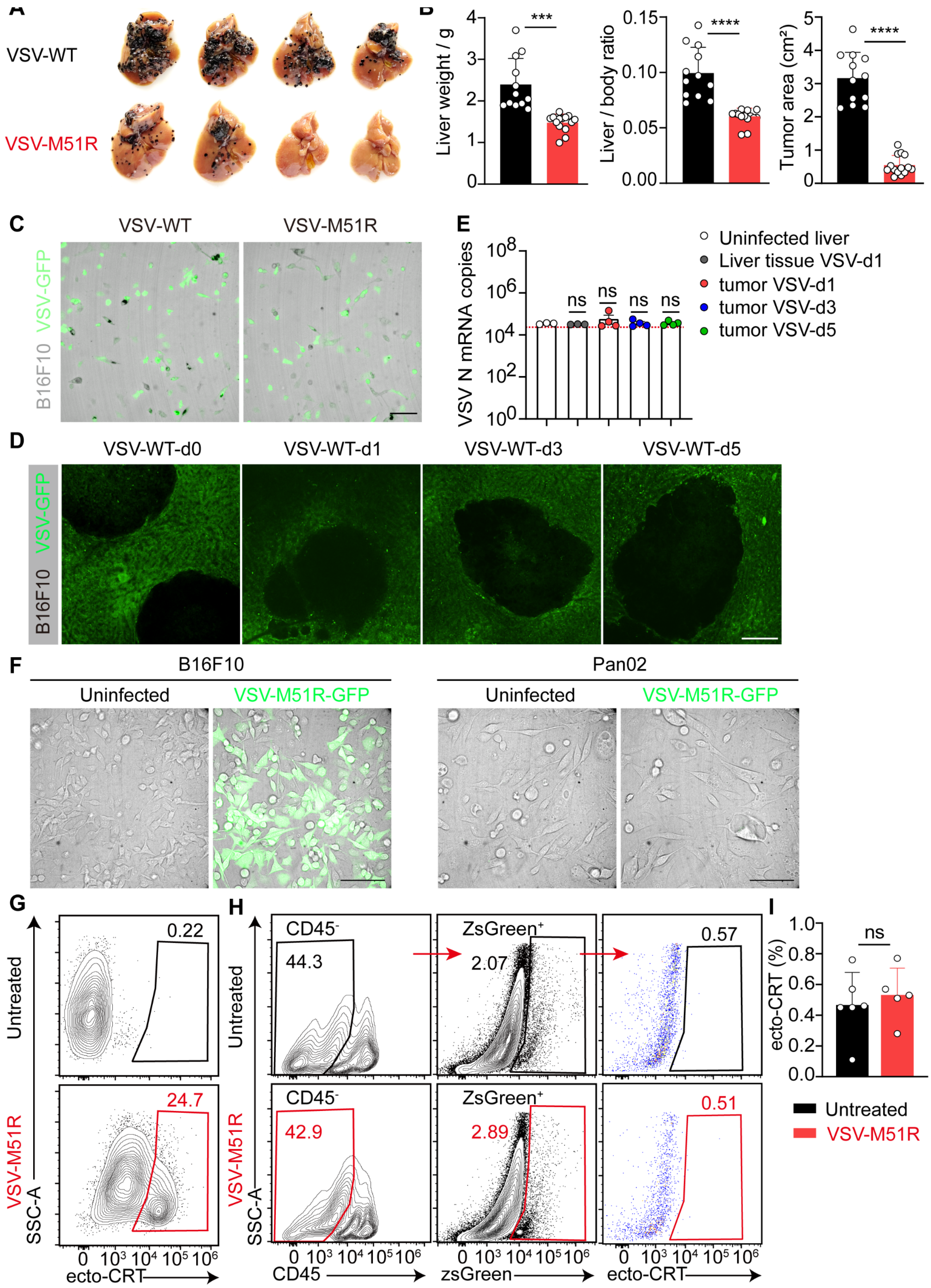

sFig. 1. VSV-M51R but not VSV-WT possesses superior therapeutic efficacy against liver metastasis independent of direct viral infection of tumor cells.

(**A**) Mice were i.v. injected with 3×10^8 PFU of VSV-WT or VSV-M51R on day 7 following B16F10 tumor inoculation and were harvested on day 14. (**B**) Liver weight, the liver-to-body weight ratio and tumor area were quantified. Data from 12 mice per

group. (**C**) Representative images showing the replication of VSV-WT-GFP or VSV-M51R-GFP in B16F10 cells *in vitro* at 24 hours post infection. (**D**) Two-photon intravital images of B16F10 liver metastases on days 1, 3, and 5 after VSV-WT-GFP treatment. Scale bar, 100 μm. (**E**) The number of VSV N mRNA copies in metastatic tumors was quantified via qPCR analysis on days 1, 3, and 5 after VSV-WT injection, and liver tissues from uninfected mice or tumor-free liver tissues from infected mice were included as controls. Data from 3–4 mice per group. Scale bar, 100 μm. (**F**) B16F10 and Pan02 cells were infected with GFP expressing VSV-M51R at a MOI of 1.2, and images were taken at 24 h post infection. (**G**) Representative flow cytometric plots of ecto-CRT expression in B16F10 cells at 24 h after VSV-M51R infection *in vitro*. (**H**) Representative flow cytometric plots of ecto-CRT expression in $CD45^-$ $ZsGreen^+$B16F10 tumor cells at 24 h after VSV-M51R treatment *in vivo*. (**I**) Quantification of the ratio of ecto-CRT^+ tumor cells in H. Data from 5–6 mice per group. Data are represented as mean ± SEM. *P < 0.05; **P< 0.01; ***P < 0.001; ns, no significance. Unpaired Student's t test for **B** and **I**, one-way ANOVA with Tukey's test for **E**.

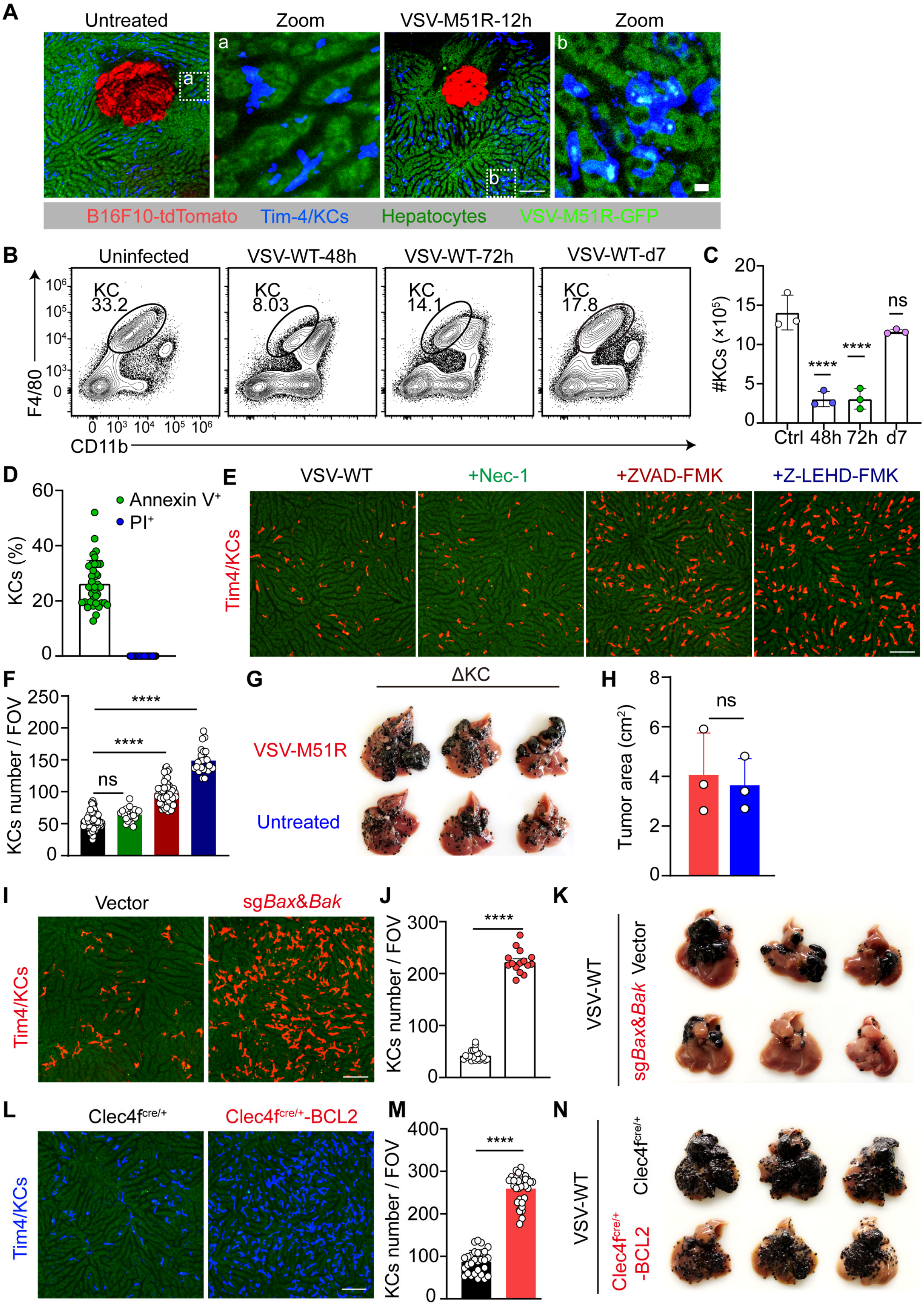

sFig. 2. Preventing KC apoptosis restores the antitumor activity of VSV-WT.

(**A**) Representative intravital liver images showing viral replication in mice bearing liver metastases at 12 hours after VSV-M51R-GFP treatment. Scale bar, 100 µm; zoom in, 10 µm. Data from 4 mice per group. (**B**) Representative flow cytometric plots and

(**C**) quantification of the percentage of KCs at the indicated time points after VSV infection. Each dot represents one mouse, n=3 mice per group. (**D**) Quantification of the ratio of Annexin V^+ or PI^+ KCs per FOV in Fig. 2K. (**E**) Representative intravital images showing KCs at 12 hours after VSV-WT infection in the absence or presence of the indicated inhibitors. Scale bar, 100 μm. (**F**) Quantification of KC numbers per FOV in E. *Clec4f-i*DTR mice were treated with DT and VSV-M51R on day 7 following B16F10 tumor inoculation. (**G**) Representative liver photos were taken on day 14, and (**H**) the tumor area was quantified. Data from 3 mice per group. (**I**) Representative intravital images of KCs at 12 hours after VSV-WT infection in mice pretreated with either Clearcoli-vector or Clearcoli-*sgBax/Bak* for *in situ* KC editing. Scale bar, 100 μm. (**J**) Quantification of KC numbers per FOV in I. (**K**) Clearcoli-vector- or Clearcoli-*sgBax/Bak*-pretreated mice were intravenously administered VSV-WT on day 7 following B16F10 tumor inoculation and were harvested on day 14. Data from 3 mice per group. (**L**) Representative intravital images showing KCs at 12 hours post VSV-WT infection in $Clec4f^{cre/+}$ or $Clec4f^{cre/+}$: LSL-hBCL2 mice. Scale bar, 100 μm. (**M**) KC density per FOV in L. (**N**) $Clec4f^{cre/+}$ or $Clec4f^{cre/+}$: LSL-hBCL2 mice were treated with VSV-WT on day 7 following B16F10 tumor inoculation and were harvested on day 14. Data from 3 mice per group. For **D**, **F**, **J** and **M**, each dot represents one FOV, with 12–24 FOVs randomly selected from 4–8 mice per group. Data are represented as mean ± SEM. $^*P < 0.05$; $^{**}P < 0.01$; $^{***}P < 0.001$, ns, no significance. Unpaired Student's t test (**D**, **H**, **J** and **M**) and one-way ANOVA with Tukey's test (**C** and **F**).

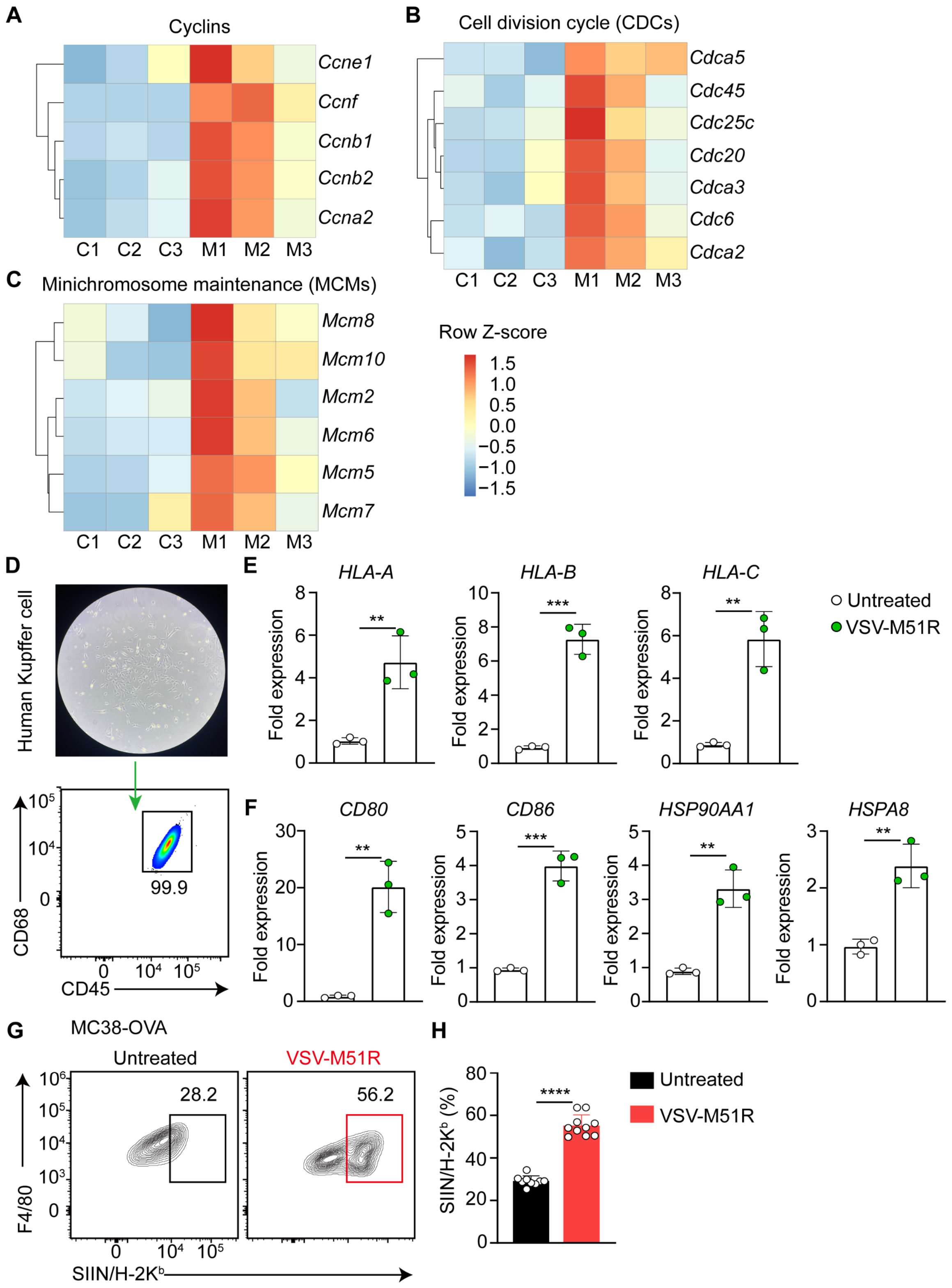

sFig. 3. VSV-M51R treatment promotes the proliferation and antigen cross-presentation of KCs.

Mice bearing B16F10 liver metastases were intravenously (iv) administered VSV-M51R for 3 days. KCs were purified from VSV-M51R-treated and untreated tumor-bearing mice and subjected to bulk RNA-seq analysis. (**A**) Heatmaps of upregulated genes

belonging to the cyclins, **(B)** the cell division cycle family (CDCs), and **(C)** the minichromosome maintenance complex family (MCM). Mice bearing MC38-OVA liver metastases were i.v. injected with VSV-M51R for 3 days. (**D**) Representative images and flow cytometric plot of human KCs at 12 h following isolation from human liver tissues. The mRNA expression levels of (**E**) *HLA-A*, *HLA-B* and *HLA-C* and (**F**) *CD80*, *CD86, HSP90AA1* and *HSPA8* in human KCs at 24 h post VSV-M51R treatment *in vitro*. Data from 3 individual cell samples per group. (**G**) Representative flow cytometric plot of SIINFEKL/H-$2K^b$ expression on KCs on day 3 following VSV-M51R treatment in metastatic MC38-OVA tumor-bearing mice. (**H**) Quantification of the proportion of SIINFEKL/H-$2K^b$ in KCs in G. Pooled data from 9–10 mice per group. Data are represented as mean ± SEM. *P < 0.05; **P < 0.01; ***P < 0.001; ns, no significance. Unpaired Student's t test.

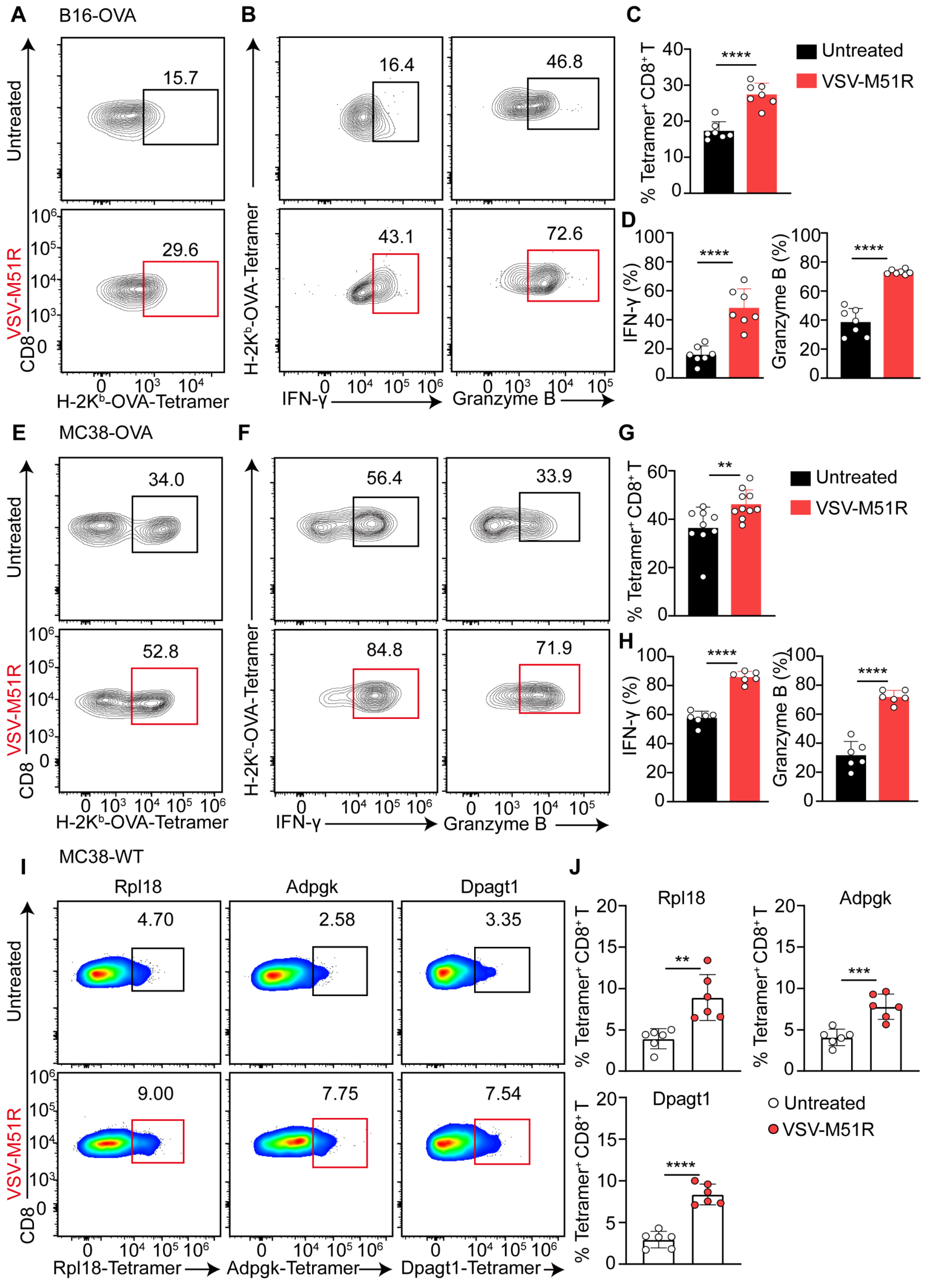

sFig. 4. VSV-M51R treatment elicits robust tumor antigen-specific CD8⁺T cell responses.

(A-D) Mice bearing B16F10-OVA liver metastases were i.v. administered VSV-M51R for 3 days. Representative flow cytometric plots of (**A**) H-2K^b-OVA tetramer$^+$ CD8$^+$ T cells and (**B**) the proportions of IFN-γ- or Granzyme B-expressing cells among

hepatic H-2K^{b}-OVA tetramer^{+} CD8^{+} T cells. (**C**) Ratio of H-2K^{b}-OVA tetramer^{+} CD8^{+} T cells in A. (**D**) Quantification of data in B; data from 7 mice per group. (E-H) Mice bearing MC38-OVA liver metastases were i.v. administered VSV-M51R for 3 days. Representative flow cytometric plots of (**E**) H-2K^{b}-OVA tetramer^{+} CD8^{+} T cells and (**F**) the proportions of IFN-γ- or Granzyme B-expressing cells among hepatic H-2K^{b}-OVA tetramer^{+} CD8^{+} T cells. (**G**) Ratio of H-2K^{b}-OVA tetramer^{+} CD8^{+} T cells in E. (**H**) Quantification in F; data from 6-10 mice per group. (I-J) Mice bearing MC38-WT liver metastases were i.v. administered VSV-M51R for 3 days. (**I**) Representative flow cytometric plots of Rpl18/Adpgk/Dpagt1 tetramer^{+} CD8^{+} T cells. (**J**) Quantification of the ratio of Rpl18/Adpgk/Dpagt1 tetramer^{+} CD8^{+} T cells in I. Data from 6 mice per group. Data are represented as mean ± SEM. *$P < 0.05$; **$P < 0.01$; ***$P < 0.001$; ns, no significance. Unpaired Student's t test.

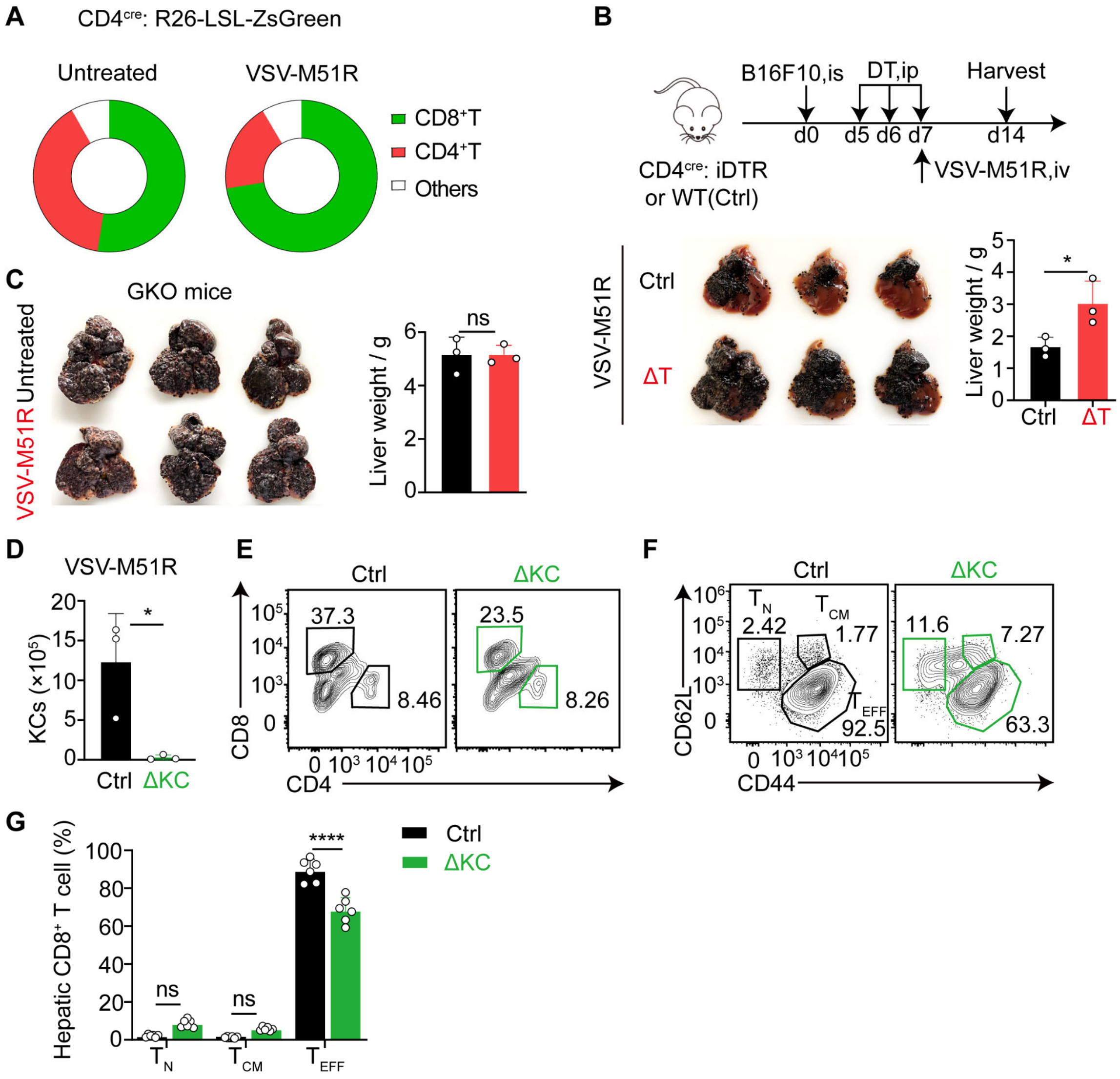

sFig. 5. VSV-M51R treatment elicits KC-dependent antitumor T-cell immunity.

(**A**) The cell composition of hepatic ZsGreen$^+$ cells from tumor-bearing *CD4^{cre}*-R26-LSL-zsGreen mice with and without VSV-M51R treatment. Data from 3 mice per group. (**B**) *CD4*- iDTR or control WT tumor-bearing mice were treated with DT or VSV-M51R as depicted, and the livers were harvested and weighed on day 14 following B16F10 tumor inoculation. Data from 3 mice per group. (**C**) Tumor-bearing *GKO* mice were treated with VSV-M51R or left untreated on day 7 post-B16F10 tumor inoculation, and the livers were harvested and weighed on day 14. Data from 3 mice per group. (D-G) *Clec4f*-iDTR or control WT mice bearing B16F10 liver metastases were treated concurrently with DT and VSV-M51R on day 7 following tumor inoculation and were harvested on day 14. **(D)** Quantification of the number of CD11b^{lo}F4/80^{hi}Tim4$^+$KCs in control or KC-depleted mice. Data from 3 mice per group. (**E**) Representative flow

cytometric plots showing the percentages of hepatic $CD8^+$ T and $CD4^+$ T cells in the control or KC-depleted groups. (**F**) Representative flow cytometric plots and **(G)** quantification of the proportions of T_N, T_{CM}, and T_{EFF} cell populations among hepatic $CD8^+$T cells. Data from 6 mice per group. Data are expressed as mean ± SEM. *P < 0.05; **P < 0.01; ***P < 0.001, ns, no significance, unpaired Student's t test (**B**, **C** and **D**), and one-way ANOVA with Tukey's test (**G**).

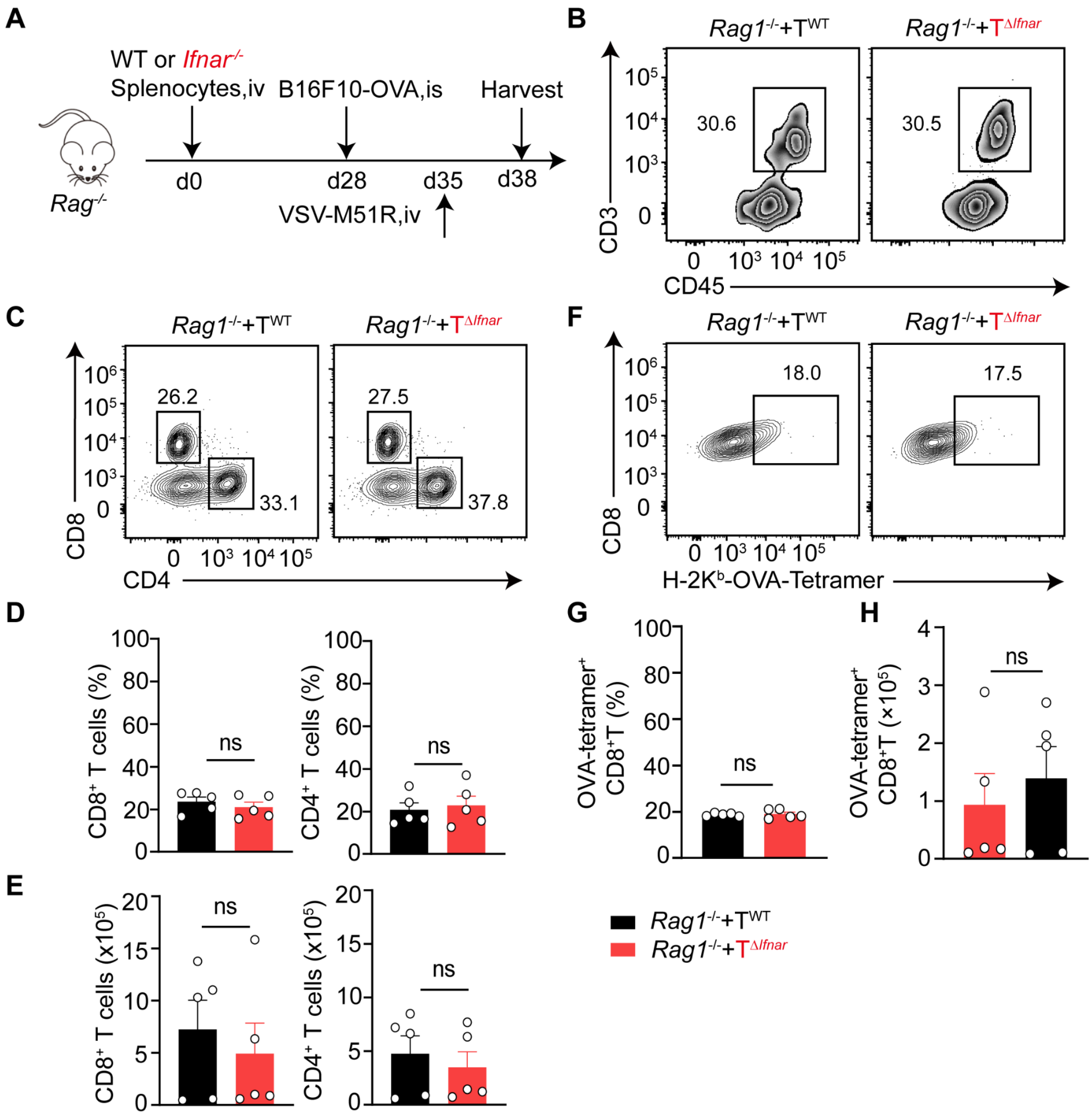

sFig. 6. Direct triggering of T cells by IFN-I is dispensable for VSV-M51R-induced antitumor CD8^{+} T-cell responses.

(**A**) Experimental design for T-cell reconstitution in *Rag1*$^{-/-}$ mice. WT or *Ifnar*$^{-/-}$ T-cell-reconstituted *Rag1*$^{-/-}$ mice were inoculated with B16F10-OVA tumors and subjected to VSV-M51R treatment as depicted. (**B**) Validating T-cell reconstitution in the blood 4 weeks after adoptive transfer. (**C**) Representative flow cytometric plots and **(D)** proportions and **(E)** numbers of hepatic CD8^{+} T and CD4^{+} T cells 3 days after VSV-M51R treatment in reconstituted *Rag1*$^{-/-}$ mice. Data from 5 mice per group. (**F**) Representative flow cytometry plots and **(G)** proportions and **(H)** numbers of hepatic SIINFEKL/H- 2K^{b} tetramer^{+} CD8^{+} T cells 3 days after VSV-M51R treatment. Data

from 5 mice per group. Data are expressed as mean ± SEM. *P < 0.05; **P < 0.01; ***P < 0.001, ns, no significance, unpaired Student's t test.

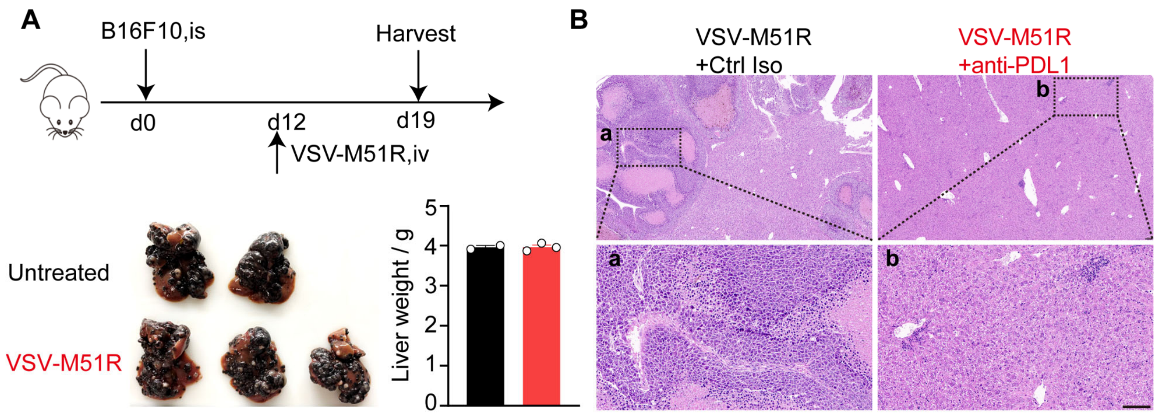

sFig. 7. Late-stage liver metastases are resistant to VSV-M51R or anti-PD-L1 monotherapy.

(**A**) Mice bearing B16F10 liver metastases were treated with VSV-M51R on day 12 posttumor inoculation and were harvested on day 19, as illustrated. n=4 mice per group, two mice from untreated group and one from VSV-M51R treated group reached the end point before day 19, and were excluded from further analysis. (**B**) Hematoxylin and eosin (H&E) staining of liver sections from VSV-M51R- or anti-PD-L1-treated B16F10 tumor-bearing mice. Scale bars, top, 100 μm; bottom, 50 μm. Data are represented as mean ± SEM.
